# *De novo* transcriptome assembly analysis of weed *Apera spica-venti* from seven tissues and growth stages

**DOI:** 10.1186/s12864-017-3538-4

**Published:** 2017-02-06

**Authors:** Marielle Babineau, Khalid Mahmood, Solvejg K. Mathiassen, Per Kudsk, Michael Kristensen

**Affiliations:** Department of Agroecology, Aarhus University, Forsøgsvej 1, Slagelse, 4200 Denmark

**Keywords:** Cytochrome P450, Glutathione S-transferase, Herbicide resistance, Loose silky bentgrass, Weed transcriptomics

## Abstract

**Background:**

Loose silky bentgrass (*Apera spica-venti*) is an important weed in Europe with a recent increase in herbicide resistance cases. The lack of genetic information about this noxious weed limits its biological understanding such as growth, reproduction, genetic variation, molecular ecology and metabolic herbicide resistance. This study produced a reference transcriptome for *A. spica-venti* from different tissues (leaf, root, stem) and various growth stages (seed at phenological stages 05, 07, 08, 09). The *de novo* assembly was performed on individual and combined dataset followed by functional annotations. Individual transcripts and gene families involved in metabolic based herbicide resistance were identified.

**Results:**

Eight separate transcriptome assemblies were performed and compared. The combined transcriptome assembly consists of 83,349 contigs with an N50 and average contig length of 762 and 658 bp, respectively. This dataset contains 74,724 transcripts consisting of total 54,846,111 bp. Among them 94% had a homologue to UniProtKB, 73% retrieved a GO mapping, and 50% were functionally annotated. Compared with other grass species, *A. spica-venti* has 26% proteins in common to *Brachypodium distachyon*, and 41% to *Lolium spp*. Glycosyltransferases had the highest number of transcripts in each tissue followed by the cytochrome P450s. The GSTF1 and CYP89A2 transcripts were recovered from the majority of tissues and aligned at a maximum of 66 and 30% to proven herbicide resistant allele from *Alopecurus myosuroides* and *Lolium rigidum,* respectively.

**Conclusions:**

*De novo* transcriptome assembly enabled the generation of the first reference transcriptome of *A. spica-venti*. This can serve as stepping stone for understanding the metabolic herbicide resistance as well as the general biology of this problematic weed. Furthermore, this large-scale sequence data is a valuable scientific resource for comparative transcriptome analysis for *Poaceae* grasses.

**Electronic supplementary material:**

The online version of this article (doi:10.1186/s12864-017-3538-4) contains supplementary material, which is available to authorized users.

## Background

Loose silky bentgrass *(Apera spica-venti* (L.) Beauv. *Poaceae: Pooidea*) occurs in Europe, Central Asia, North-West Africa, Caucasus, Turkey, and North-West Iran [1]. It is the most serious agricultural weed in Eastern and Central Europe, infesting many crops particularly winter wheat and winter barley [2–4]. It is an annual, dioecious, outcrossing species with bisexual flowers. Its life cycle consists of seed germination in autumn, then overwintering at the 2-3 leaf stage. It resumes growth in early spring becoming taller than most crop species. It flowers in mid-summer and finally sheds seeds around July [5]. One plant can produce up to 16,000 seeds which enables it to infest whole fields within a few generations [2]. At a density of 200 plants per m^2^, *A. spica-venti* decreases the yield of winter wheat up to 30% while another study [5] estimates the reduction in crop yield to be proportional to the quantity of the weed [2]. The European populations have shown a high degree of morphological and genetic variation [6] allowing it to adapt quickly to changing agricultural landscapes.

Control of *Apera spica-venti* has been, and still is, heavily relying upon herbicides, specifically acetolactate synthase (ALS) and acetyl-CoA (ACCase) inhibitors [7]. However, the continuous use of herbicides favored the evolution of resistance in *A. spica-venti* populations. Resistance to ALS herbicides was reported for the first time in Switzerland in 1994, and has since been found in 9 other European countries [7]. The study of resistance mechanisms is dependent on previous knowledge of genetic information such as gene identification, abundance, and nucleotide sequences. Information on other genes not related to herbicide resistance, as well as genetic information from susceptible genotypes, are, however, necessary for proper investigations [8].

Transcriptome data have been used in different weed species to study the origin of polyploidy events [9, 10], to understand genetics and biology of weeds [11–14], and to investigate herbicide resistance [15–22]. The majority of transcriptomic studies have focused on dictoyledonous weed species and only four grass weeds have been investigated at the transcriptomic level: *Lolium rigidum* [22, 23], *Eleusine indica* [24], *Poa annua* [15, 25], and *Echinocloa cruss-galli* [16]. The investigation into evolutionary and molecular processes in these weeds is therefore facilitated by the availability of molecular data. Publicly available genetic information on the diploid species *A. spica-venti* is very limited: only 12 nucleotide sequences from five genes (*ALS*, *ACCase*, *rbcL*, *trnL* and *matK*) are available in the NCBI database [26]. Eight of these come from phylogenetic studies while the four remaining sequences are from herbicide resistance studies [27–29].

The aim of this study was to establish a *de novo* assembled comprehensive reference transcriptome of *A. spica-venti* from different tissues (leaf, root, and stem) and early phenological growth stages BBCH05, 07, 08, and 09. We used multiple individuals from various herbicide susceptible populations in order to represent a precise and accurate reference transcriptome for this species which would contain the genetic variation within this outcrossing species. Each dataset, as well as the combined dataset, were *de novo* assembled and compared among themselves and to similar dataset from the grass weed *Lolium rigidum* and the model organism *Brachypodium distachyon*. Known herbicide resistance gene families, cytochrome P450 monooxygenase (P450), glutathione S-transferase (GST), ATP-binding cassette transporters (ABC), elongation initiation factor (EIF), transcription factor (TF) and glycosyltransferase (GT), were identified and transcript abundance was quantified in each tissue.

## Results

### Transcriptome assembly

A *de novo* assembly approach was used in order to obtain a comprehensive reference transcriptome of *A. spica-venti.* The Illumina sequencing resulted in eight libraries having between 54 to 80 × 10^6^ reads each with an average read length of 145 bp for a total dataset of 80,885 Mbp. On average, >80% of the raw reads passed the quality control and normalization process with an average percentage of bases quality score >30 (%Q30) of 88%, an average phred score of 37 with mean base call accuracy of 99.99%. After trimming, quality control and normalization, 13 to 18 × 10^6^ high quality paired-end reads remained in the different libraries. These were assembled into contigs ranging from 32,001 to 83,349 (Table 1). A *de novo* assembly using Trinity software resulted in a contig number between 117,629 and 319,916 (Table 1). These were passed to the redundancy reduction steps (longest isoform selection, 90% sequence similarity merging and translation into protein sequence using longest ORF: see Methods). The combined assembly show transcript lengths between 99 and 200 bp, while 657 transcripts are longer than 1,000 bp (Additional file 1). Finally, between 28,000 and 43,000 unigenes were identified in the eight individual non-redundant assemblies. Of these, >95% were annotated to UniprotKB and to *Brachypodium distachyon* coding sequences (Table 1). Some assembly statistics are lower for the combined assembly (N50, average contig lenght) compared to the individual assemblies, some statistics are higher or similar (GC%, protein coding transcripts, max contig lenght).

**Table 1:** Assembly and annotation statistics for the combined assembly containing all three tissues and four growth stages along with individual assemblies for the seven tissue/growth stages of *A. spica-venti*

	Seed 05	Seed 07	Seed 08	Seed 09	Leaf	Root	Stem	All tissues
Surviving Paired Reads	33,047,108	34,553,732	35,507,768	34,083,440	27,518,992	47,701,648	28,792,480	241,205,168
Transcript number before rr^a^	2,300,488	319,916	284,019	266,532	149,469	222,747	117,629	669,871
Transcripts number after rr^a^	41,963	45,511	43,324	47,423	32,001	46,168	33,244	83,349
Genes	38,088	42,292	40,042	43,316	28,246	38,972	28,513	74,724
Total bases	33,259,794	31,598,526	31,320,780	34,985,208	24,432,147	36,146,334	29,060,739	54,846,111
GC %	53	53	53	53	52	53	53	53
Number of protein coding transcripts								
> = 1 kbp	9,887	8,024	8,628	9,873	7,207	10,922	9,803	13,019
Max contig lenght	11,865	10,923	11,733	9,633	11,736	14,148	12,282	14,247
average contig lenght	776	694	722	737	763	782	874	658
N50	999	834	900	927	966	1,017	1143	762
N50 longest isoform	1,038	849	915	951	1,014	1,098	1,218	783
BLASTp:								
hit % *B. distachyon*	97	98	98	97	98	97	98	97
hit % Uniprot	96	96	97	96	96	95	96	94
% GO mapped	72	73	74	74	75	74	73	73
% functionally annotated	56	58	58	56	62	55	58	50

^a^rr; redundancy reduction steps (after the Trinity de novo assembly). Surviving paired reads represents paired reads remaining after the quality control and normalization steps

Similar to the individual assemblies, the combined dataset has 10,163 sequences that have an enzyme code representing 12% of the total transcript number. The e-values distribution of the combined assembly shows the 78% of transcripts with an e-value of <1e^−10^ (Additional file 2) indicating high confidence in the annotation performed

### Transcriptome assembly quality

The eight assemblies show a high percentage of completeness (Table 2), both when comparing them to all plants using Benchmarking Universal Single-Copy Orthologs (BUSCO) and Reads Mapped Back to the Transcriptome (RMBT) where paired-end reads are aligned to their respective transcriptome assembly. For example, the combined assembly is 83% complete and 90% of its reads mapped back to its own transcripts. This indicates that these assemblies are properly assembled and are of good quality. The percentage of reads from each library that are properly paired when mapped to the combined assembly (RMBT to Full in Table 2) is greater than the percentage properly paired when mapped to the assembly from that library (RMBT to themselves in Table 2) indicating that our combined assembly represent well the individual assemblies. Intermediate levels of duplication within assemblies (18-24%) were found in the BUSCO analysis (Table 2).

**Table 2:** Quality assessment of the assemblies using Benchmarking Universal Single-Copy Orthologs (BUSCO) and Reads Mapped Back to the Transcriptome (RMBT) of themselves and the combined assembly

	Seed 05	Seed 07	Seed 08	Seed 09	Leaf	Root	Stem	All tissues
BUSCO (%):								
Completeness	86	76	79	83	74	84	84	83
Duplicated	18	18	19	20	18	18	17	24
Fragmented	5	16	13	9	13	7	8	10
Missing	7	7	7	7	11	7	7	5
RMBT to themselves:								
% mapped	86.1	96.3	87.6	86.6	88.8	85.6	86.2	90.9
% properly paired	75.9	87.5	76.6	75.9	81.0	76.8	77.9	80.1
% singletons	3.8	1.3	3.4	3.7	3.4	3.9	3.6	3.2
Total bp	16,523,554	17,276,866	17,753,884	17,041,720	13,759,496	23,850,824	14,396,240	120,602,584
RMBT to Full:								
% mapped	95.5	96.3	96.0	95.4	97.2	94.5	97.4	95.9
% properly paired	86.2	87.5	87.4	86.3	89.9	86.2	89.0	87.3
% singletons	1.4	1.3	1.3	1.5	1.0	1.4	1.0	1.3
Total bp	16,523,554	17,276,866	17,753,884	17,041,720	13,759,496	23,850,824	14,396,240	120,602,584

**Fig. 1:**
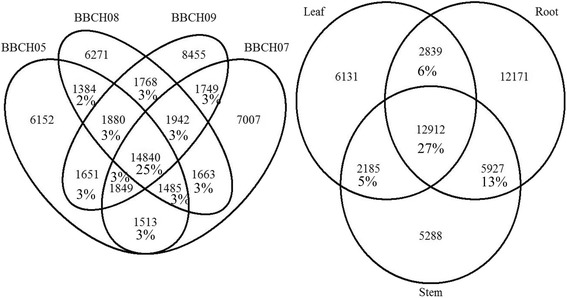
Venn diagram of non-redundant proteins. Left, between the four seed growth stages BBCH05, 07, 08, and 09. Right, between the three tissues leaves, roots, and stems

This observed level of redundancy led us to create a zero-redundancy protein ID list for each assembly in order to compare the similarity between the different assemblies without the redundancy. We wanted to ensure that the level of sequence duplication was not introducing a bias in the comparative analysis of the assemblies. The comparison of zero-redundant protein IDs with their respective original assemblies reveals a 24, 27, 25, 25, 22, 23 and 18% of redundancy in seed BBCH05, 07, 08, 09, leaves, root and stem, respectively. These are similar levels of redundancy as was observed with BUSCO. The zero-redundant protein lists were further used to compare the number of shared proteins between tissues and growth stages (Fig. 1). The four germinated seed stages share 25% of proteins, while the three tissues share 27% of proteins (Fig. 1). The percentage of shared proteins between the 3 seed stages are lower (3%) indicating that each growth stage is genetically distinct. The assemblies for roots and stems are more similar than to the leaves assembly. Each tissue/stage has a large number of unique proteins. Root assembly has the highest proportion of unique proteins with 12,171 sequences representing 36% of all its non-redundant expressed proteins recovered.

### Functional annotation

**Fig. 2:**
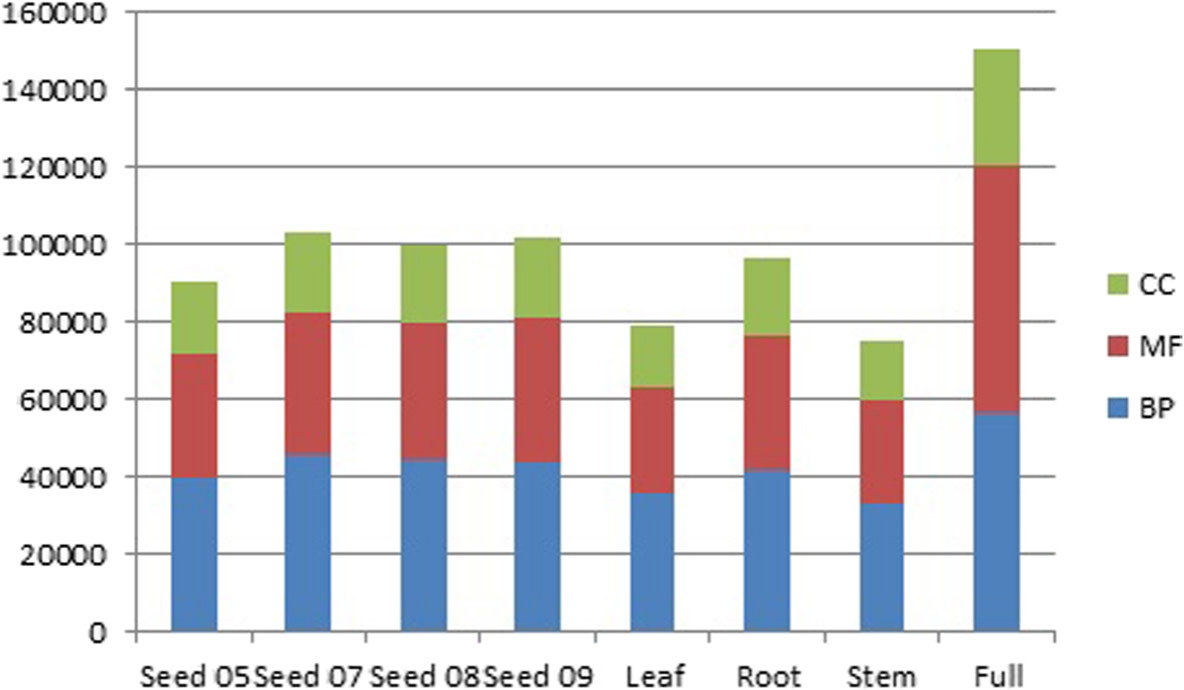
Comparison of Gene Ontology terms found in the seven individual and combined assemblies. BP; biological processes, MF; metabolic function, CC; cellular component

The lack of functional molecular data regarding *A. spica-venti* prompted us to perform a functional annotation on all eight assemblies. Similar to the individual assemblies, the combined dataset has 10,163 sequences that have an enzyme code representing 12% of the total transcript number. The e-values distribution of the combined assembly shows that 78% of the transcripts were annotated with an e-value of <1e^−10^ (Additional file 2) indicating high confidence in the annotation performed. The combined assembly has 56,716 sequences belonging to the gene ontology (GO) category of biological process, 63,685 to metabolic function, and 30,063 to cellular component for a total of 150,464 GO annotations (Additional file 3). The “organic substance metabolic process” was the most represented within the category Biological Process (BP) with 15,792 transcripts, then “ion binding” for the Metabolic Function (MF) category with 18,318, and “intracellular function” for the cellular component (CC) with 9,168 transcripts. The comparison of functional annotations between individual assemblies shows that these are rather similar in their GO annotations (Fig. 2). There does not appear to be any disproportionally represented GO categories (BP, MF, CC) between the different tissues and seed growth stages (Fig. 2).

Kyoto Encyclopedia of Genes and Genomes (KEGG) analysis can be used to identify potential pathways most represented in each tissue/growth stages of *A. spica-venti*. The top ten KEGG pathways, based on sequences number, were compared between each assembly. Purine metabolism was the pathway with the most sequences in each individual assembly with 1,206, 1,400, 1,348, 1,324, 1,061, 1,309, 1,031 and 1,932 sequences in seeds BBCH05, 07, 08, 09, leaves, stem, root and combined assembly, respectively. Carbon fixation in photosynthetic organisms was also observed in the top ten for seeds at BBCH07, 08, 09, and in leaves with 116, 136, 118, and 108 sequences, respectively. All assemblies have similar pathways in their top ten such as important metabolisms pathways (pyruvate, pyrimidine, methane) and sugar pathways (starch and sucrose biosynthesis, amino and nucleotide sugar biosynthesis, and glycolysis and gluconeogenesis). These most represented pathways are important basic pathways necessary for cell life and therefore found in high amounts in all plants. In the top ten KEGG pathways, biosynthesis of antibiotics was recovered in all assemblies while the plant secondary metabolite pathway was not recovered in any assembly. In order to assess for fungal or bacterial contamination in our assemblies, every homologue species recovered from the annotation was verified and confirmed as either algae or green plant organisms. Secondly, the annotation of twenty random sequences that were categorized as part of the biosynthesis of antibiotics was verified. The twenty sequences were all annotated to grass plants with a similarity percentage greater than 70%. We believe that many plant secondary metabolites were most likely defined as antibiotics (such as phytoalexins) in the KEGG database. Our results indicate that plant antibiotics are produced to a large extent in all tissues of *A. spica-venti*.

The GO enrichment analysis revealed no significantly enriched GO category between individual assemblies and the combined assembly. This result is consistent with Figs. 1 and 2, indicating that the individual assemblies are well represented within the combined and that the individual assemblies are similar to each other.

### Comparison to other grass species

**Fig. 3:**
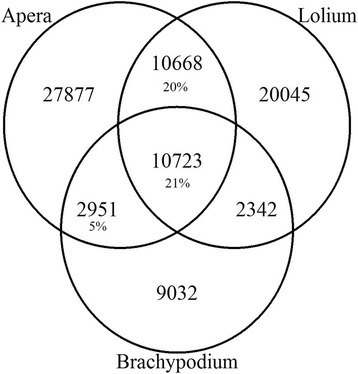
Venn diagram of non redundant proteins between *A. spica-venti, Lolium*, and *B. distachyon*

Comparative approaches are effective for finding differences and analogies between molecular dataset. Sequence conservation of the *A. spica-venti* assembly to phylogenetically related grasses and weeds was thus performed. Following annotation to UniprotKB, the highest numbers of homologues for the combined assembly were found in *Aegilops tauschii* with 10,266 transcripts representing 12% of the species distribution, followed by *Triticum aestivum* with 8,990 transcripts for 11% of transcripts (Additional file 4). Overall, the top twenty species account for 77% of homologues in the combined assembly. The individual assemblies showed a similar species distribution (data not shown). A comparison of non-redundant protein ID was used (similar to above) between *A. spica-venti*, *Lolium spp.* and *B. distachyon*. The *Lolium spp.* sequences used come from Schliesky et al. 2012 [30] and originate from a concatenation of three species (*L. perenne, L. multiflorum*, and *L. rigidum*). The annotation of the *Lolium spp.* transcriptome yielded 86,417 annotated protein sequences of which 48,251 (56%) were non-redundant. *Brachypodium distachyon* yielded 32,086 protein transcripts of which 25,048 (78%) were non-redundant. *Apera spica-venti* had 53,005 (58%) non-redundant protein annotations. These three species share 21% proteins (Fig. 3). *Apera* has a higher percentage of protein similarity to *Lolium* (41%) than to *B. distachyon* (26%). *Apera* also has a similar annotation redundancy percentage to *Lolium spp* with 42% and 44%, respectively. Twentytwo percentage of annotations in *B. distachyon* was redundant. This lower number is expected considering that this species is a model organism with better annotated data and also because the sequences used here are *ab initio* predicted from cDNA extracted from genomic studies.

### Herbicide resistance gene family

**Fig. 4:**
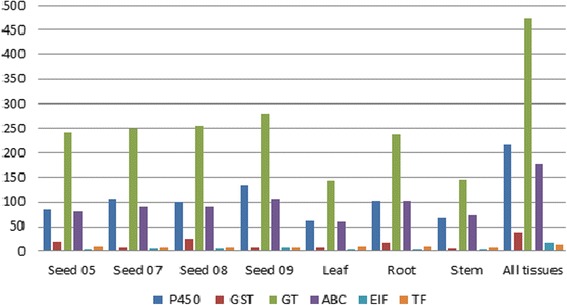
Transcript abundance for major herbicide resistance gene families in the seven different tissues and growth stages from *A. spica-venti*. P450; cytochrome P450, GST; glutathione S-transferase, GT; glycosyltransferase, ABC; ABC transporters, EIF; elongation initiation factor, TF; transcription factor

Genes involved in herbicide resistance mechanisms were identified in varying number in the different tissues of *A. spica venti.* Glycosyltransferase is the most abundant (Fig. 4) in each tissue and growth stage. Seed growth stages had the highest number of GT (excluding the combined assembly) with an average of 250 sequences versus 170 sequences for the three tissues (Fig. 4). The majority of GT sequences found were homologues of barley (*Hordeum vulgare* var. *distichum*). The GST transcripts were found in low amount in *A. spica-venti* overall (Fig. 4). Five sequences identified as GSTF1 in *Apera* were identified from seeds BBCH05, BBCH07, BBCH08, root and stem. Sequences identified as GSTF1 were aligned with the AmGSTF1 identified in blackgrass (*Alopecurus myosuroides*; Additional file 5) as this specific sequence was shown to endow herbicide resistance [31]. These had a 38-66% similarity to the *A. myosuroides* AmGSTF1 sequence. This large range of percentage of similarity is attributable to length differences as no SNPs were identified between the different *Apera* GSTF1 sequences. However, a three amino acids insertion was found in the blackgrass sequence. The three amino acids are Asparagine, Glutamine and Valine (DEV) at position 104 to 106 bp (Additional File 5). Both alleles are functional. These amino acids were not present in the *Apera* neither in the *Triticum uratu* sequence. It is possible that this insertion in *A. myosuroides* is actually a deletion in *A. spica-venti* and *T. uratu*. A GST1 identified in *E. crus-galli* also responsible for herbicide resistance [32] was also identified in *Apera*.

**Fig. 5:**
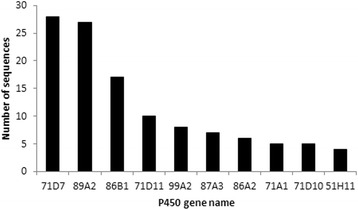
Top 10 most abundant cytochrome P450s in *A. spica-venti*

**Fig. 6:**
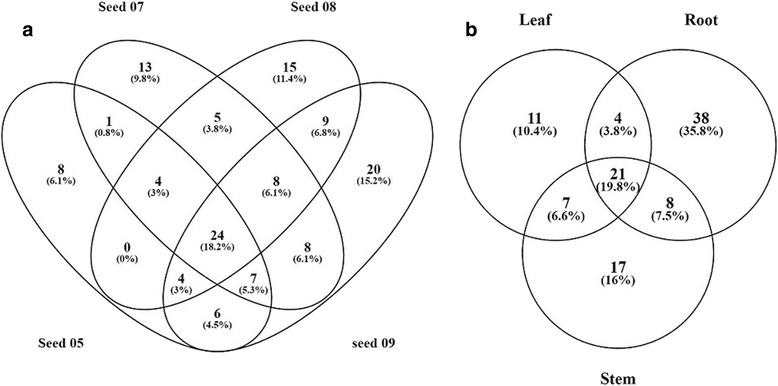
Venn diagram of shared P450 proteins. *Left*, between germinated seeds at BBCH stage 05, 07, 08, and 09. *Right*, between leaf, root, and stem

The cytochrome P450s transcripts were found in high numbers across *A. spica-venti* tissues and growth stages (Fig. 4). We identified a total of 85, 107, 100, 133, 61, 102 and 67 cytochrome P450 sequences in seeds BBCH 05, 07, 08, 09, leaves, root and stem respectively (Fig. 4). A total of 49 different cytochrome P450s were identified throughout the seven individuals assemblies. Many sequences were identified as P450s but did not have a subfamily identification and therefore were not counted in this number. The most abundant P450 was from subfamily CYP71 (CYP71D7) with 28 sequences identified as such in the combined assembly (Fig. 5). This specific P450 was also the most abundant of the P450s in the seed stages and in leaves with 15, 16, 10, 17 and 6 sequences respectively. The second most abundant P450 is CYP89A2 (Fig. 5). Sequences identified as CYP89A2 in *A. spica-venti* were aligned with the CYP89A2 identified in *L. rigidum* (Additional file 6) shown to confer herbicide resistance [22]. Multiple CYP89A2 sequences (mostly from *T. uratu* and *Aegilops tauschii*) were identified in each tissue, with the highest number (13) found in seeds at BBCH 05. Overall, 58 sequences of P450 CYP89A2 were found. A large variation in length was observed with the shortest being 99 amino acid long and the longest 488 amino acids (average: 208 aa). The alignment of all CYP89A2 sequences show a 14-40% sequence similarity to *Lolium spp*, with the highest score belonging to sequences that were annotated to the gene TRIUR3_23510 (Uniprot ORF number) from *T. uratu.* This demonstrates high sequence polymorphism among the four grass species for CYP89A2. The P450 family was further compared based on protein identification between the seven tissues and growth stages. Overall, the four seed growth stages express 18% of the same P450 in optimal growing conditions (Fig. 6). Seeds at the BBCH 09 had the highest number of uniquely expressed P450s (Fig. 6). The three tissues have almost 20% of identical P450s expressed while root have the highest number of uniquely expressed P450s.

## Discussion

The aim of this study was to enhance genetic resources for *A. spica-venti* and through the provision of a reference transcriptome creating a platform to decipher, among others, the development of herbicide resistance. In this study, through a *de novo* assembly and redundancy reduction strategy, 74,724 unigenes were found in the combined assembly of *A. spica-venti* which showed a high level of completeness, as well as in the seven individual assemblies. The combined assembly is expected to have assembly statistics differences with the individual assemblies since it contains much more reads and has a 7-fold duplication of many transcripts.

The assembly and homology search statistics are all congruent with previous transcriptome studies in *Poaceae*. The observed N50 for *A. spica-venti* are similar to the values from *Lolium spp de novo* transcriptome (771 bp; [23]), *L. rigidum* (1,150: [22]), *Leymus chinensis* (813: [33]) and *Phragmites australis* (1,187 bp; [34]), but are lower when compared to *Zea mays* (1,612 bp :[30]), and *Arundo donax* (2,229 bp ; [35]), *Poa annua* (1,602 bp: [25]), *L. perenne* (1,500 bp: [36]), and *Eleusine indica* (2,095 bp: [24]). The number of transcript after redundancy reduction is slightly lower than the number found after filtering in *Lolium spp.* [23]. The percentage of functionally annotated protein sequence is also in accordance with *Lolium spp.* results (57%: [25]). The observed top GO terms in each category (BP, CC, MF) are also found in top positions in *E. indica* [24], *L. chinensis* [33], and *E. crus-galli* [16]. The percentage of properly paired reads to their respective assembly (RMBT) is in line with similar results from *A. donax* [34]. Overall, the similarity found between the *A. spica-venti* transcriptome and other grass transcriptomes indicate a properly assembled and annotated dataset presented here.

Although a strict redundancy reduction strategy was used during the assembly, the *A. spica-venti* transcriptome still shows an intermediate level of transcript redundancy. This indicates that many reconstructed transcript were similar enough to annotate to the same description, yet different enough not to be combined during the assembly. Several biological and technical biases could explain this. Biologically, different gene copy number, alternative splicing, heterozygosity and transcripts from large gene families could explain intermediate levels of redundancy. Alternatively, library preparation such as the amplification step and RNA fragmentation steps, as well as fragmented contigs could also be an explanation. This redundancy can be seen in the alignment of CYP89A2 (see below), where there is a large variation in sequence similarity and length. Both *A. spica-venti* and *Lolium spp* showed similar levels of redundancy. This high level of sequence heterogeneity most likely stems from the heterogeneous genetic background of multiple individuals from different populations used in this study to create the transcriptome representing natural variation within *A. spica-venti*. Transcript redundancy is a biological artifact that complicates *de novo* transcriptome assembly when using heterogeneous data [23]. Experimentally, contig redundancy could be minimized by performing several generational crosses between individuals from the same population. The level of redundancy in *A. spica-venti* is consistent across tissues and growth stages and can be identified by multiple methods presented here (BUSCO, non-redundant protein ID) and most importantly, does not affect the levels of similarity between *A. spica-venti* assemblies. With and without the redundancy, the *A. spica-venti* transcriptome has high levels of similarity with phylogenetically related grass species such as *B. distachyon* and *Lolium spp*. Our study shows that a reference transcriptome can be successfully created from multiple genetically heterogeneous individuals. Using extra steps after the initial *de novo* assembly can reduce redundancy drastically.

The absence of GO enrichment between the different tissues and growth stages is unsurprising considering the large sample size being compared and the fact that the assemblies were constructed from material under the same biotic and abiotic conditions. This lack of enrichment indicates also that the combined assembly represents well the variation within each tissue and growth stages. The fact that the *A. spica-venti* transcriptome is more similar to *Lolium spp.* than to *B. distachyon*, despite *B. distachyon* being a much better annotated reference, is most likely caused by the close phylogenetic relationship or could indicate a genetic similarity between grass weeds. The genera *Apera, Lolium, Alopecurus* and *Poa* are all part of the large monophyletic core *Pooideae* while *Brachypodium* is part of the Brachypodieae [37].

Many populations of *A. spica-venti* are resistant to herbicides and some have been identified as metabolic resistance cases, with a strong suspicion on the cytochrome P450s family, but never confirmed [38]. We find a higher number of total sequences corresponding to some key herbicide resistance gene families in *A. spica-venti* than compared to *Lolium rigidum* reference, which contained 56 GST and 7 GT sequences [22]. The total number of different P450s recovered in this study (49) is in line with the 57 recovered in the transcriptome of *Lolium rigidum* [22]. However, this number is far below the number of P450s identified in other Poaceae species such as rice (228) and sorghum (326), respectively [39]. Transcriptome studies do not recover the total number of genes present in an organism. This tells us that herbicide susceptible populations of *A. spica-venti* in Denmark express a low number of P450s in optimal growing conditions. This is consistent with the knowledge of P450s as being involved in stress and xenobioticide defense in plants. The fact that the roots presented the largest number of unique P450s could indicate they were under some undetermined stress and more likely to indicate higher basal P450 expression. Roots in *Zea mays* and in *Arabidopsis* are known to express P450s [40, 41] and some P450s are expressed predominantly in roots [42], but there is no evidence that this is a general trend for the P450 family. The most abundant P450, CYP71D7, has been shown to be implicated in glycoalkaloid and ginsenosides biosynthesis, stress-induced secondary metabolites, as well as green tissue regeneration, especially in shoot development stages, in many crops [43–46]. Interestingly, CYP71D7 is also highly upregulated in blight-resistant potato [47]. This hints at a potential role in herbicide resistance in *A. spica-venti*, but remains to be investigated.

The large sequence variation in CYP89A2 between *Lolium*, *Apera*, and *Triticum*, could indicate that these sequences are not highly conserved as they are implicated in environmental adaptation. The *A. spica-venti* and *A. myosuroides* GSTF1 sequences are similar indicating a potential for similar metabolic activity in the presence of herbicide as was demonstrated in Cummins et al [31]. These two known resistance genes (CYP89A2 and GSTF1) were recovered in the majority of tissues of this reference transcriptome of *A. spica-venti* indicate that these are constitutively expressed at many early stages and overall in the plant. GST are known to be expressed at every stage of plant development in every tissue [48]. Many of the known herbicide resistant P450s, GSTs, and EIFs identified in other weeds species [22, 23, 49–53] were not recovered.

## Conclusion

In the present study, we have described the construction and functional annotation of a *de novo* reference transcriptome assembly for *Apera spica-venti* with emphasis on gene families involved in herbicide detoxification. These transcriptomes represents a considerable addition to the molecular knowledge available for this important weed species. Moreover, GO and KEGG analysis were conducted and all unigenes were classified into functional categories with the aim to understand their role and metabolic pathways. This data can be used to develop oligo-nucleotide microarray to study gene expression studies at large scale. We have characterized the major gene families responsible for herbicide resistance and shown strong potential of two specific genes to have a role in herbicide resistance in *A. spica-venti*. Finally, this study can serve as a reference to unravel genes and pathways involved in metabolic herbicide resistance mechanisms, weed adaption, grass genetics and trait evolution in the future.

## Methods

### Tissue collection

Seeds from six herbicide susceptible populations of *Apera spica-venti* originating at six different locations in Denmark, were mixed together to create a meta-population encompassing a range of genetic variations possible within the species. Each of these six populations were previously tested between 2007 and 2010 with ALS and ACCase inhibitor herbicides and found to be susceptible (Mathiassen, pers. comm). Seeds were sown in pots and grown under greenhouse conditions on a table with an automatic watering system. Leaf, stem, and root material from individual plant were harvested once the plants were at BBCH stage 34 to 36 (stem elongation). Tissues were cut in small pieces and immediately put in liquid nitrogen and then stored at -80 °C. These tissues were kept separated by individual plant. Seeds from this meta-population were sown in 9 cm petri dishes with four filter papers and 5 mL of deionized water and grown in a climate cabinet (Termaks Series KB8000L) at night/day temperatures of 10 °C/17 °C for 10 h/14 h. Germinated seeds were harvested at stage BBCH 05 (radicle emerges from seed), 07 (coleoptile emerges from caryopsis), 08 (coleoptile elongation), and 09 (Emergence: coleoptile breaks through soil surface). Germinated seeds were put in RNAlater RNA stabilization reagent (Qiagen GmbH, Hilden, Germany) and then stored at 4 °C.

### RNA extraction and cDNA sequencing

A total of 0.5 g of frozen tissue from leaf, stem, and root was grounded separately using a 2010 Geno/Grinder (SPEX Sample prep, Stanmore, UK) for 45 s at 1,500 Hz and then soaked in liquid nitrogen. A total of 0.7 g of germinated seeds were grounded using the same method but for 2 cycles of 45 s at 1,500 Hz and were not soaked in liquid nitrogen. The RNA mini plant kit (Qiagen, Hilsen, Germany) was used for RNA extraction following the manufacturer protocol, three wash steps were performed. RNA quality was verified using a spectrophotometer (Nanodrop 3300, Wilmington, USA) and bioanalyzer (Agilent 2100, Santa Clara, USA). Samples that showed a RIN value of 6.0 and more were treated with DNAse 1 according to the protocol (Qiagen, Hilsen, Germany) and suspended in a final volume of 14 μL. RNA quality was verified again with spectrophotometer (NanoDrop-1000 v.3.1.0) and bioanalyzer using the RNA 6000 Plant Nano program (Agilent 2100). Samples with a RIN value higher than 6 were selected for cDNA library construction. The lower than recommended (>8) RIN value threshold selected here is because of difficulties in extracting high quality RNA from germinated seed samples. High quality RNA from minimum three individuals was pooled for each tissue and seed growth stages. Nine libraries were created; seven for each of the individual tissues or growth stage, and libraries for leaf and root, respectively. Samples of mRNA were selected, fragmented and transformed to 150-400 bp short insert, strand specific cDNA libraries for sequencing on Illumina HiSeq 2500 (Eurofins MGW, Germany).

### *De novo* transcriptome assembly of *Apera spica-venti*

The raw reads were quality-clipped before performing the assembly using the software Trimmomatic [54]. Using a sliding window approach, raw reads with a Phred score below 20, containing only “N”, and/or length below 80 bp were removed. These were normalized using kmer size of 20 [55]. Transcript reconstruction is computationally challenging, especially for non-model plant species [30]. The de Bruijn’s graph strategy has shown to be the best performing for Illumina reads [56]. The Trinity assembler was selected as it has been shown to be more accurate than Velvet to reconstruct full length transcriptome [57]. A combined dataset was created by merging all reads from individual libraries. The assembly method was a composite of different methods in order to reduce transcripts redundancy. First the *de novo* Trinity pipeline (version 2.1.1; [58]) was used with a minimum contig length of 200 bp and a minimum kmer covariance of 2. The longest isoform for each gene was selected (perl script by Brian Haas on trinityrnaseq-users google group), then contigs were merged according to a similarity criterion of 90% in CD-HIT-EST (version 4.6.3, [59]). The contigs were then translated to coding protein sequences using Transdecoder (version 2.0.1; [56]) following identification of the longest ORFs. This pipeline was performed for each of the seven tissue- and growth stage- specific libraries. Non-redundant assemblies were used in downstream functional annotation KEGG, and GO enrichment analysis. Raw reads, assembled and annotated data set are available at NCBI Gene Expression Omnibus; submission GSE86989.

### Assembly quality

The quality of the assemblies was assessed using three methods. First, peptide sequences from the longest ORF from each library were blasted (blastp) against *Brachypodium distachyon* peptide sequences (Ensembl) as this species is the closest model plant species to *A. spica-venti*. Second, the assemblies were evaluated against a database of single copy orthologue genes for plants as implemented in BUSCO (version 1.161; [60]). Thirdly, the number of reads that mapped back to the combined transcriptome assembly, and to their own respective assembly, was assessed (RMBT; [61]). Comparing the number of unique proteins IDs recovered (no redundancy) to the original protein list allowed us to calculate a redundancy percentage.

### Functional annotation, KEGG and GO enrichment

Individual libraries and the combined, were each aligned (blastp v.3.2.2) to the non-redundant plant protein database UniprotKB (viridiplantae 75_2015-11) with an e-value filter of 1e^−1^ and only the best homologue was reported. GO mapping was performed against the Gene Ontology database implemented in B2G (version 3.3.5; [62]). Annotation was performed with an e-value hit filter set to 1e^−1^, annotation cutoff of 55, and evidence code set to 0.8 for the different categories as implemented in B2G. The KEGG pathways was performed in B2G based on enzyme code in each assembly. GO enrichment analysis was performed using a two sided Fisher Exact Test with the multiple testing corrections of Benjamini and Hochberg (FDR) of 0.05. The combined assembly was used as the reference while each annotated assemblies were tested individually against it.

### Homology to other model grass species

Protein sequences from *Lolium spp.* and *Brachypodium distachyon* were aligned by blastp (e-value filter of 1e^−1^ and only the best homologue was reported) to the same version of UniprotKB database used for *A. spica-venti.* This was done in order to compare the three species based on exactly the same search parameters and database type and version. *B. distachyon* is the closest model species, while *Lolium* is a genus of grass weed with a well-characterized transcriptome [22, 23, 36]. The coding sequences used for *Lolium spp.* were taken from the recent work of Duhoux et al. [30] which was generated using tissues from the three species of rye-grasses (*L. perenne, L. multiflorum*, and *L. rigidum*). Similarly to the *A. spica-venti* transcriptome generated here which contains different individuals from field conditions, the *Lolium spp.* transcriptome contains a high level of heterogeneity stemming from different field individuals and from different species within the *Lolium* genus. The number of identical non-redundant proteins recovered between *Apera, Lolium*, and *Brachypodium* was compared, as well as redundancy percentage.

### Herbicide resistance gene families

The number of transcripts annotated to known herbicide resistance gene families (P450, GST, GT, TF, ABC and EIF were compared between the seven assemblies, and also to *B. distachyon* genome and *L. rigidum* transcriptome. Specific transcripts identified to genes conferring herbicide resistance in previous studies were aligned to assess identity, homology, and potential resistance phenotype in *A. spica-venti*. Because the majority of resistance genes identified in herbicide resistant weeds are P450s, this gene family was further investigated. The top ten most abundant P450s recovered were identified. The number of P450s common between tissues and growth stages were compared.

## Additional files

Additional file 1: Sequence length distribution for combined assembly. (JPG 25 kb)
Additional file 2: E-value distribution for combined assembly. (PNG 9 kb)
Additional file 3: Plant Gene Ontology terms associated with the combined assembly of all tissues and growth stage of *A. spica-venti*. BP; biological processes, MF; metabolic function, CC; cellular component. (PNG 34 kb)
Additional file 4: Top 20 species identification for the combined dataset. (JPG 74 kb)
Additional file 5: Alignment of cytochrome P450s CYP89A2 protein sequences from *Apera, Lolium, Triticum*, and *Aegilops*. (RTF 1753 kb)
Additional file 6: Alignment of GSTF1 protein sequences from *Apera* and *Alopecurus*. (RTF 159 kb)

## Abbreviations

ABC: ATP-binding cassette transporters
ACCase: Acetyl-CoA
ALS: Acetolactate synthase
BBCH: Bundesanstalt Bundessortenamt und Chemische Industrie
BP: Biological process
BUSCO: Benchmarking universal single-copy orthologs
CC: Cellular component
EIF: Elongation initiation factor
GO: Gene ontology
GST: Glutathione S-tranferase
GT: Glycosyltransferase
KEGG: Kyoto Encyclopedia of Genes and Genomes
MF: Metabolic function
NTSR: Non-target site resistance
P450: Cytochrome monooxygenase P450
RMBT: Reads mapped back to the transcriptome
TF: Transcription factor
