# Supplementary figures and images for: De novo transcriptome assembly analysis of weed Apera spica-venti from seven tissues and growth stages
Source: BMC Genomics. 2017 Feb 6;18:128. doi: 10.1186/s12864-017-3538-4 (PMC5294808; doi:10.1186/s12864-017-3538-4)

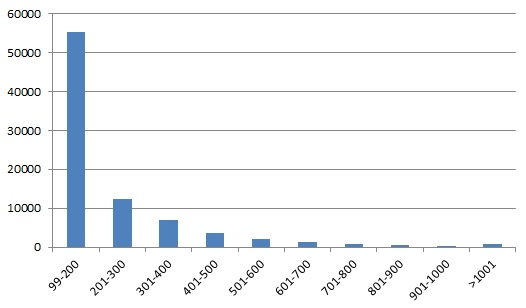

Supplement: Additional file 1: — Sequence length distribution for combined assembly. (JPG 25 kb) [file 12864_2017_3538_MOESM1_ESM.jpg]

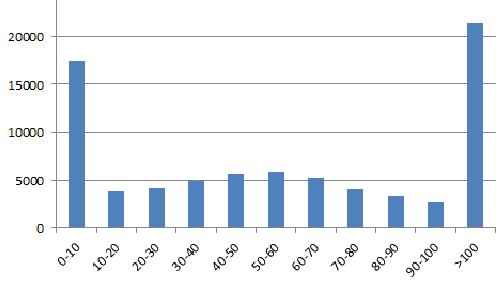

Supplement: Additional file 2: — E-value distribution for combined assembly. (PNG 9 kb) [file 12864_2017_3538_MOESM2_ESM.png]

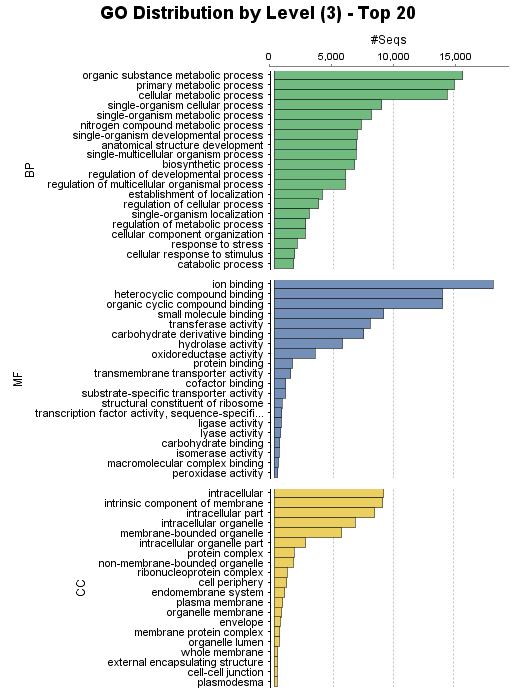

Supplement: Additional file 3: — Plant Gene Ontology terms associated with the combined assembly of all tissues and growth stage of A. spica-venti. BP; biological processes, MF; metabolic function, CC; cellular component. (PNG 34 kb) [file 12864_2017_3538_MOESM3_ESM.png]

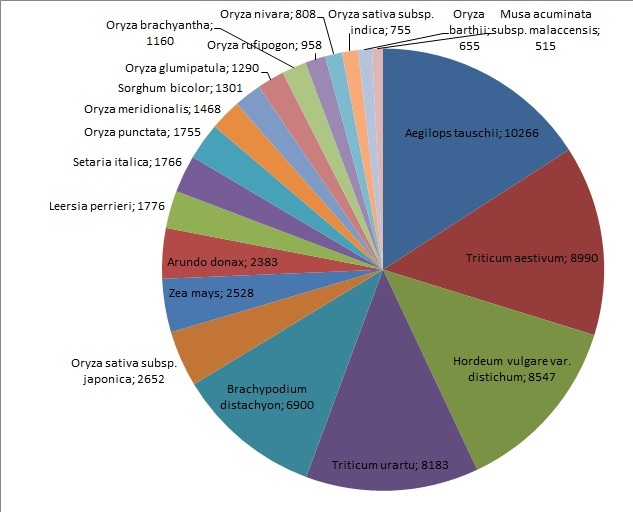

Supplement: Additional file 4: — Top 20 species identification for the combined dataset. (JPG 74 kb) [file 12864_2017_3538_MOESM4_ESM.jpg]
