# Supplementary material for: De novo transcriptome assembly analysis of weed Apera spica-venti from seven tissues and growth stages
Source: BMC Genomics. 2017 Feb 6;18:128. doi: 10.1186/s12864-017-3538-4 (PMC5294808; doi:10.1186/s12864-017-3538-4)
Supplement: Additional file 5: — Alignment of cytochrome P450s CYP89A2 protein sequences from Apera, Lolium, Triticum, and Aegilops. (RTF 1753 kb) [file 12864_2017_3538_MOESM5_ESM.rtf]

Additional file 5. Alignment of cytochrome P450s CYP89A2 protein sequences from Apera, Lolium, Triticum, and Aegilops.

                                         10        20        30        40        50        60        70        80        90       100                  
                                ....|....|....|....|....|....|....|....|....|....|....|....|....|....|....|....|....|....|....|....|
Arapidopsis_gi|15217771|ref|NP  MEIWLLILASLSGSLLLHLLLRRRNSSSPPLPPDPNFLPFLGTLQWLREGLGGLESYLRSVHHRLGPIVTLRITSRPAIFVADRSLTHEALVLNGAVYAD 
M8a6n8                          MGDPRFYLCRESTFEPAIFIAGRDAAHRTLARDGATFAHRPPSWSFGFNAHGVNSAQYGGRWSLLRRNISSHLAGAPLAGALQYSVGKLVSSLECAAAAA 
M7zhu4                          MADLLLLMLPLALLFFLLHHASPIKALLTRAKSVLAPELSTVWKPQPASIFVTDHETAHSLLIQNTLPMVSAGAPFVTTSHRRSFTRRGSTGTRRHAAGI 
M8axb5                          MEFMLIVLLLSLVLCISAVAVFRRRAWAWAVHDGQQPIIEIHDPTIARRALMDHADAFCNRPLNLFPVALVSGRRRSHSDNLTSVPYGPRWRALRCTLNA 
M7z1d9                          MELLVLIALLLLGLTVLIRRRSYIYTTSTSRARAPAAVVIQRIADPAVAHRALVENADDFSDRPVAPFLVFLEKKHGQYGDGLASAPYGPLWRAFRCNIT 
M8CS19|M8CS19_AEGTA Cytochrome  MQDLLVLTLSLMLLLVAMVVRRHGHASSKAVYTRLATSLKSTFAGWLRQPAIVIRDRATAHRLLVRGCVGGSFSNRPASMAPSSVLSHRRYHNLTSAPYG 
M8A0V3|M8A0V3_TRIUA Cytochrome  ------------------------------------MELVVLLVVVLLGFSVLIRRSSRRAPAPPAPAVLHKISDPAVAHRVLVDNADAFSNRPVLPFFV 
M8A8M2|M8A8M2_TRIUA Cytochrome  ---MDELTILLCIALSIPLIIFCMLKRHGGRQKLPPGPPALLFIAKLLLMLKSPIYHLGPVLRSLHARYGPIVSVCLGRTFVFVADRHLTHSALVKGGGN 
M7ZAM7|M7ZAM7_TRIUA Cytochrome  -----------------------------------------------------------------MDTWHIFLGSILLAIPLILLLRGRKGGRLRRGATL 
Lolium_Gaines_89A2              WQSMEVLNILLCIILSLP--LIFLLS-HHGRKKLPPGPPTLLFIAK-FLLPGRSASHIGPMLRGLHARHGPIFSFWLLRMFVFVDDRHLTHSVLVKGAAT 
A2_TRINITY_DN68910_c0_g1_i3|m.  ---------------------------------------------------------------------------------------------------- 
A2_TRINITY_DN66356_c0_g2_i1|m.  -------------------------LAGPKPPSIDISMDLLVLLALLLLVGLSVFIRRSSSSPRRALAPAPPAVHKISDPGVAHRVLVAFSDRPFLPFFV 
A2_TRINITY_DN68910_c0_g2_i1|m.  ---------------------------------------------------------------------------------------------------- 
A2_TRINITY_DN70484_c0_g3_i1|m.  ------------------------------------------------------------------------------------ADRRLTHSTLIKGGGN 
A2_TRINITY_DN73066_c0_g3_i1|m.  ---------------------------------------------------------------------------------------------------- 
A2_TRINITY_DN77876_c5_g6_i1|m.  ---------------------------------------------------------------------------------------------------- 
A2_TRINITY_DN88769_c1_g2_i1|m.  ---------------------------------------------------------------------------------------------------- 
A2_TRINITY_DN88769_c2_g3_i3|m.  ---------------------------------------------------------------------------------------------------- 
A2_TRINITY_DN88769_c2_g6_i2|m.  ---------------------------------------------------------------------------------------------------- 
A2_TRINITY_DN91187_c2_g16_i2|m  ---------------------------------------------------------------------------------------------------- 
A3_TRINITY_DN101639_c0_g4_i1|m  ---------------------------------------------------------------------------------------------------- 
A3_TRINITY_DN101639_c0_g5_i1|m  ---------------------------------------------------------------------------------------------------- 
A3_TRINITY_DN101639_c0_g6_i1|m  ---------------------------------------------------------------------------------------------------- 
A3_TRINITY_DN113586_c9_g10_i1|  ---------------------------------------------------------------------------------------------------- 
A3_TRINITY_DN35959_c0_g1_i1|m.  ---------------------------------------------------------------------------------------------------- 
A3_TRINITY_DN47294_c0_g2_i1|m.  ---------------------------------------------------------------------------------------------------- 
A3_TRINITY_DN70793_c0_g2_i1|m.  ---------------------------------------------------------------------------------------------------- 
A3_TRINITY_DN80133_c0_g1_i1|m.  ---------------------------------------------------------------------------------------------------- 
A3_TRINITY_DN93016_c2_g1_i2|m.  -----------------------------------------------------------------------------------HRVLVAFSNRPVLPFFV 
A3_TRINITY_DN93016_c1_g2_i1|m.  ---------------------------------------------------------------------------------------------------- 
A4_TRINITY_DN105332_c2_g3_i2|m  ---MDGLTILLSLTLSIP--LVILMLNRHGRKNLPPGPPALVFIAK-MLAYGWPIYQFGPVLRSLHARYGPVISVCLGQTFVFVADRRLTHSTLIKGGGN 
A4_TRINITY_DN39512_c0_g1_i1|m.  ---------------------------------------------------------------------------------------------------- 
A4_TRINITY_DN42484_c0_g1_i1|m.  ---------------------------------------------------------------------------------------------------- 
A4_TRINITY_DN76031_c0_g1_i1|m.  ---------------------------------------------------------------------------------------------------- 
A4_TRINITY_DN76068_c0_g3_i1|m.  ---------------------------------------------------------------------------------------------------- 
A4_TRINITY_DN77267_c1_g3_i1|m.  ---------------------------------------------------------------------------------------------------- 
A4_TRINITY_DN86750_c1_g4_i1|m.  ---------------------------------------------------------------------------------------------------- 
A4_TRINITY_DN86750_c1_g5_i1|m.  ---------------------------------------------------------------------------------------------------- 
A4_TRINITY_DN88254_c3_g1_i1|m.  -----------------------------------------------------------------------------------HRVLVAFSNRPVLPFFV 
A6_TRINITY_DN58600_c1_g3_i1|m.  ---------------------------------------------------------------------------------------------------- 
A6_TRINITY_DN66770_c1_g1_i1|m.  ---------------------------------------------------------------------------------------------------- 
A7_TRINITY_DN126267_c0_g1_i1|m  ---------------------------------------------------------------------------------------------------- 
A7_TRINITY_DN163078_c0_g1_i1|m  ---------------------------------------------------------------------------------------------------- 
A7_TRINITY_DN73089_c0_g1_i1|m.  ---------------------------------------------------------------------------------------------------- 
A7_TRINITY_DN73089_c1_g3_i1|m.  ---------------------------------------------------------------------------------------------------- 
A7_TRINITY_DN87364_c0_g1_i1|m.  -------------------------------------MDLLVLLALLLFGGFSVFIHRSSS--RRAPAPAPPAVHKISDPAAAHRVLVAFSDRPFLPFFV 
A7_TRINITY_DN88932_c0_g2_i1|m.  ---------------------------------------------------------------------------------------------------- 
A7_TRINITY_DN94510_c4_g1_i2|m.  ---MDGLTILLCITLSIP--LIILMVNRHGRKNLPPGPPALVFIAK-MLAYGWPIYQFGPVLRSLHARYGPVISVCLGQTFVFVADRRLTHSTLIKGGGN 
A7_TRINITY_DN94510_c4_g2_i1|m.  ---------------------------------------------------------------------------------------------------- 
A7_TRINITY_DN94953_c0_g1_i1|m.  ---------------------------------------------------------------------------------------------------- 
A5_TRINITY_DN102218_c5_g6_i2|m  ---------------------------------------------------------------------------------------------------- 
A5_TRINITY_DN103684_c0_g2_i1|m  ---------------------------------------------------------------------------------------------------- 
A5_TRINITY_DN103684_c0_g5_i1|m  ---------------------------------------------------------------------------------------------------- 
A5_TRINITY_DN103684_c1_g3_i3|m  ---------------------------------------------------------------------------------------------------- 
A5_TRINITY_DN34162_c0_g1_i1|m.  ---------------------------------------------------------------------------------------------------- 
A5_TRINITY_DN81309_c2_g1_i1|m.  -------------------------VAGPFPTTMEQ---LLVLLALFLLGGFSVLIRRSSS--RRAPAPAPPAVHTISDPAVAHRVLVAFSNRPVLPFFV 
A5_TRINITY_DN81309_c2_g2_i2|m.  -------------------------------------MDLLVLLALLLLVGLSVFIRRSSSSPRRALAPAPPAVHKISDPGVAHRVLVAFSDRPFLPFFV 
A5_TRINITY_DN88739_c0_g1_i1|m.  ---------------------------------------------------------------------------------------------------- 
A5_TRINITY_DN89190_c1_g7_i1|m.  ---------------------------------------------------------------------------------------------------- 
A5_TRINITY_DN93741_c1_g1_i1|m.  ---------------------------------------------------------------------------------------------------- 
A5_TRINITY_DN97327_c1_g3_i2|m.  ---------------------------------------------------------------------------------------------------- 
A8_TRINITY_DN28752_c0_g1_i1|m.  ---------------------------------------------------------------------------------------------------- 
A8_TRINITY_DN33033_c0_g1_i1|m.  ---------------------------------------------------------------------------------------------------- 
A8_TRINITY_DN40046_c1_g1_i1|m.  ---------------------------------------------------------------------------------------------------- 
A8_TRINITY_DN42043_c0_g1_i1|m.  -------------------------------------MDLLVLLALLLFGGFSVFIHRSSS--RRAPAPAPPAVHKISDPAAAHRVLVAFSDRPFLPFFV 
A8_TRINITY_DN49523_c2_g1_i1|m.  ---------------------------------------------------------------------------------------------------- 
A8_TRINITY_DN52955_c1_g4_i1|m.  ---MDGLTILLCVTLSIS--LIIFLLNRPGRQNLPPGPPSLLFVAK-MLTLKSPIYHFGPVLRSLHARYGPIVSVCLGRTFVFVADRRLTHSTLIKGGGN 
A8_TRINITY_DN52955_c1_g5_i1|m.  ---MDGLTILLCITLSIP--LIILMLNRHGRKNLPPGPPALVFIAK-MLAYGWPIYQFGPVLRSLHARYGPVISVCLGQTFVFVADRRLTHSTLIKGGGN 
A8_TRINITY_DN52955_c1_g6_i1|m.  ---------------------------------------------------------------------------------------------------- 

                                        110       120       130       140       150       160       170       180       190       200         
                                ....|....|....|....|....|....|....|....|....|....|....|....|....|....|....|....|....|....|....|....|
Arapidopsis_gi|15217771|ref|NP  RPPPAVISKIVDEHNISSGSYGATWRLLRRNITSEILHPSRVRSYSHARHWVLEILFERFRNHGGEEPIVLIHHLHYAMFALLVLMCFGDKLDEKQIKEV 
M8a6n8                          KNHVVVPSEMLRHAVVSFFASLCFGEGVEEDVLRQLRGVHAEILSLVVELGAFHLMPALLEVACYFPRCRKLSNVQKRHRATVMTLISARQQRDSDGVGS 
M7zhu4                          ATMCFGDGIDNTHVRVMADAQSDLVKSLATARVFAHAKLPAVTRFIYRNRWKKLAALRQQQEETYLPLIDSRRGRHRQPSETPVTQGGIEEGCPTVNSWA 
M8axb5                          AVLHPSRLGHTDPLRLETMDALVADLRVRCVGGGGEIVVRDILNAAVFPLVARMCFGSGVDEGHVRAMRDLLQAFVLAVDGSKDFASSKTAKLLHWRQWR 
M7z1d9                          SETLHPSRLGHVTPLQREAIHGLVANLGKELPVVTVVRDHLYPSIFSLLARLCFGDDVDEGHVRVMGCLIREFQQVAVGEARASPGTMLAKLAEWRRLRR 
M8CS19|M8CS19_AEGTA Cytochrome  PFWRVARRNLTSEVLHPVRLHRYAAARRDALCGLVADLREQCTSKPDGLVLSAESIRTAMFGLLTTMCFGEGVDAGLTRAMADAQHDLVQFFPELRVFAK 
M8A0V3|M8A0V3_TRIUA Cytochrome  ELAKRRGGQRNENISTVPHGPHWRALRCNITAETLHPSRLGLGHLAPLQREAIQDLVAALQSTGAGAG--------LRDHLRAAVFRVIARLCFGDG-VD 
M8A8M2|M8A8M2_TRIUA Cytochrome  FDTRPPPSKLFTLFLGGSMSASPLGPYFRLVRRNLHSQALHPSRVRLLAPVRQRVCDALVGSLRRDRDAASQGTVTVRPLMARCLFDLLTSMTFGVTLGQ 
M7ZAM7|M7ZAM7_TRIUA Cytochrome  ADRPALPSLKLLGENNNTITRASYGPVWRLLRRNLVSETLHPSRVRLFAPARSWVRRVLAEKLRDESSSGGAAVVET---FQYAMFCLLVLMCFGERLDE 
Lolium_Gaines_89A2              STADHRRTGLCNSFFPHGIGTSPYGDYWRLLRRNLNVHALHPSRIRLLEPARQRARNAMLASLRADVGADASRVITVRPFLARCLFQLIVEMSLGARLGQ 
A2_TRINITY_DN68910_c0_g1_i3|m.  ---------------------------------------------------------------------------------------------------- 
A2_TRINITY_DN66356_c0_g2_i1|m.  ELAKRHGGRRNDNVGTAAYGPLWHALRRNMTAETFHPS--RLAQLAPLQREAIQGLVAALQSAGGAAAACRVEVDNLRDHLRAALFRLVARMCFGDGTVD 
A2_TRINITY_DN68910_c0_g2_i1|m.  ---------------------------------------------------------------------------------------------------- 
A2_TRINITY_DN70484_c0_g3_i1|m.  FDTRPPPSEMFELFLGGSMAASPLGAYFRLVRRNLHSQALHQSRVRLFAPARQRICAAFVGSLRRDRDAASDGIVTVRPSLARCLFSLLTCMCFG----- 
A2_TRINITY_DN73066_c0_g3_i1|m.  -------------------------------------QALHPSRVRLFAPARQRVCDAFVGSLRRDRDAASQGIVTVRPLMSRCLFDLLTCMTFGVTLGQ 
A2_TRINITY_DN77876_c5_g6_i1|m.  ---------------------------------------------------------------------------------------------------- 
A2_TRINITY_DN88769_c1_g2_i1|m.  ---------------------------------------------------------------------------------------------------- 
A2_TRINITY_DN88769_c2_g3_i3|m.  ------------------------------LRRNLVAETLHPSRVRLFAPARAWVRRVLAEKLRERDDDSSAPVLET---FQYAMFCLLVLMCFGERLDE 
A2_TRINITY_DN88769_c2_g6_i2|m.  ---------------------------------------------------------------------------------------------------- 
A2_TRINITY_DN91187_c2_g16_i2|m  ---------------------------------------------------------------------------------------------------- 
A3_TRINITY_DN101639_c0_g4_i1|m  -----------------SMAASPLGAYFRLVRRNLHSQALHQSRVRLFAPARQRICAAFVGSLRLDRDAASDGIVTVRPSLARCLFSLLTCMCFGVTLGQ 
A3_TRINITY_DN101639_c0_g5_i1|m  -------------------------------PPHPALAAAAPVPRQALRAARQRVFDAFVDSLRRDRDAASQGIVTVRALMSRCLFDLLTSMTFGVTLGQ 
A3_TRINITY_DN101639_c0_g6_i1|m  ------------LYLGGSMSASPLGPYFRQVRRTLHSQALHPSRVMLFAPARQRVFDAFVDSLRRDRDAASQGIVTVRALMSRCLFDLLTCMTFGVTLGQ 
A3_TRINITY_DN113586_c9_g10_i1|  ---------------------------------------------------------------------------------------------------- 
A3_TRINITY_DN35959_c0_g1_i1|m.  ---------------------------------------------------------------------------------------------------- 
A3_TRINITY_DN47294_c0_g2_i1|m.  ---------------------------------------------------------------------------------------------------- 
A3_TRINITY_DN70793_c0_g2_i1|m.  ---------------------------------------------------------------------------------------------------- 
A3_TRINITY_DN80133_c0_g1_i1|m.  ---------------------------------------------------------------------------------------------------- 
A3_TRINITY_DN93016_c2_g1_i2|m.  ELAKRQGGRRNDNIGTATYGPHWHALRPSLTAETLHPS--RLGQLAPLQQDATEDLVAALQSAGGAAG-----VDNIRDHLRAAVFRVVARLCFGDG-VD 
A3_TRINITY_DN93016_c1_g2_i1|m.  -----------------------------------------------------------LQSAGGAAAACRVEVDNLRDHLRAALFRLVARMCFGDGTVD 
A4_TRINITY_DN105332_c2_g3_i2|m  FDTRPPPSEMFELFLGGSMAAS------------------------------------------------------------------------------ 
A4_TRINITY_DN39512_c0_g1_i1|m.  ---------------------------------------------------------------------------------------------------- 
A4_TRINITY_DN42484_c0_g1_i1|m.  ---------------------------------------------------------------------------------------------------- 
A4_TRINITY_DN76031_c0_g1_i1|m.  ---------------------------------------------------------------------------------------------------- 
A4_TRINITY_DN76068_c0_g3_i1|m.  ----------------------SYGSVWRLLRRNLVAETLHPSRVRLFAPARAWVRRVLSEKLRD-DSAGGAAVVES---FHYAMFCLLVLMCFGELLDE 
A4_TRINITY_DN77267_c1_g3_i1|m.  ---------------------------------------------------------------------------------------------------- 
A4_TRINITY_DN86750_c1_g4_i1|m.  ---------------------------------------------------------------------------------------------------- 
A4_TRINITY_DN86750_c1_g5_i1|m.  ---------------------------------------------------------------------------------------------------- 
A4_TRINITY_DN88254_c3_g1_i1|m.  ELAKRQGGRRNDNIGTATYGPHWHALRRSLTAETLHPS--RLGQLAPLQQDATEDLVAALQSAGGAPG-----VDNIRDHLRAAVFRVVARLCFGDG-VD 
A6_TRINITY_DN58600_c1_g3_i1|m.  ------------------------------------------------------------------------------------------------MDNE 
A6_TRINITY_DN66770_c1_g1_i1|m.  ---------------------------------------------------------------------------------------------------- 
A7_TRINITY_DN126267_c0_g1_i1|m  ---------------------------------------------------------------------------------------------------- 
A7_TRINITY_DN163078_c0_g1_i1|m  ---------------------------------------------------------------------------------------------------- 
A7_TRINITY_DN73089_c0_g1_i1|m.  ---------------------------------------------------------------------------------------------------- 
A7_TRINITY_DN73089_c1_g3_i1|m.  ---------------------------------------------------------------------------------------------------- 
A7_TRINITY_DN87364_c0_g1_i1|m.  ELAKRHGGRRNDNVGTAAYGPLWHALRRNMTAETFHPS--RLAQLAPLQREATQGLVAALQSAGAR------RVDNLRDHLRAALFRVVARMCFGDGTVD 
A7_TRINITY_DN88932_c0_g2_i1|m.  ---------------------------------------------------------------------------------------------------- 
A7_TRINITY_DN94510_c4_g1_i2|m.  FDTRPPPSEMFELFLGGSMAASPLGAYFRLVRRNLHSQALHQSCVRLFAPARQRICAAFVGSLRRDRDAASDGIVTVRPSLARCLFSLLTCMCFGVTLGQ 
A7_TRINITY_DN94510_c4_g2_i1|m.  ----------------------------------------------------QRICAAFVGSLRRDRDAASDGIVTVRPLMSRCLFDLLTCMTFGVTLGQ 
A7_TRINITY_DN94953_c0_g1_i1|m.  ---------------------------------------------------------ALLRKVQQPPVLVVRDRVAAHRLLVRGSAGGCFSDRPASTAGS 
A5_TRINITY_DN102218_c5_g6_i2|m  ---------------------------------------------------------------------------------------------------- 
A5_TRINITY_DN103684_c0_g2_i1|m  -------------------------------RRTLHSQALHPSRVRLFAPARQRVFDAFVDSLRRDRDAASQGIVTVRALMSRCLFDLLTCMTFGVTLGQ 
A5_TRINITY_DN103684_c0_g5_i1|m  ---------------------------------------------------------------------------------------------------- 
A5_TRINITY_DN103684_c1_g3_i3|m  ---------------------------------------------------------------------------------------------------- 
A5_TRINITY_DN34162_c0_g1_i1|m.  ----------------------------------------------------------------------------------------------SPRCQE 
A5_TRINITY_DN81309_c2_g1_i1|m.  ELAKRQGGRRNDNIGTATYGPHWHALRRSLTAETLHPS--RLGQLAPLQQDATEDLVAALQSAGG----------------------------------- 
A5_TRINITY_DN81309_c2_g2_i2|m.  ELAKRHGGRRNDNVGTAAYGPLWHALRRNMTAETFHPS--RLAQLAPLQREAIQGLVAALQSAGGAAAACRVEVDNLRDHLRAALFRLVARMCFGDGTVD 
A5_TRINITY_DN88739_c0_g1_i1|m.  -------------------------------------------------PARQRICAAFVGSLRRDRDAASDGIVTVRPSLARCLFSLLTCMCFGVTLGQ 
A5_TRINITY_DN89190_c1_g7_i1|m.  ---------------------------------------------------------------------------------------------------- 
A5_TRINITY_DN93741_c1_g1_i1|m.  ---------------------------------------------------------------------------------------------------- 
A5_TRINITY_DN97327_c1_g3_i2|m.  ---------------------------------------------------------------------------------------------------- 
A8_TRINITY_DN28752_c0_g1_i1|m.  ---------------------------------------------------------------------------------------------------- 
A8_TRINITY_DN33033_c0_g1_i1|m.  ---------------------------------------------------------------------------------------------------E 
A8_TRINITY_DN40046_c1_g1_i1|m.  ---------------------------------------------------------------------------------------------------- 
A8_TRINITY_DN42043_c0_g1_i1|m.  ELAKRHGGRRNDNVGTAAYGPLWHALRRNMTAETFHPS--RLAQLAPLQREATQGLVAALQSAGAR------RVDNLRDHLRAALFRVVARMCFGDGTVD 
A8_TRINITY_DN49523_c2_g1_i1|m.  ---------------------------------------------------------------------------------------------------- 
A8_TRINITY_DN52955_c1_g4_i1|m.  FDTRPPPSKIFTLYLGGSMSASPLGPYFRQVRRTLHSQALHPSRVRLFAPARQRVCDAFVGSLRRDRDAASDGIVTVRPLMSRCLFDLLTCMTFGVTLGQ 
A8_TRINITY_DN52955_c1_g5_i1|m.  FDTRPPPSEMFELFLGGSMAASPLGAYFRLVRRNLHSQALHQSCVRLFAPARQRICAAFVGSLRRDRDAASDGIVTVRPSLARCLFSLLTCMCFGVTLGQ 
A8_TRINITY_DN52955_c1_g6_i1|m.  ----------------------------------------------------------------------------------YAAVFAMVARMCFGDGVG 

                                        210       220       230       240       250       260       270       280       290       300         
                                ....|....|....|....|....|....|....|....|....|....|....|....|....|....|....|....|....|....|....|....|
Arapidopsis_gi|15217771|ref|NP  EFIQRLQLLSLTKFNIFNIWPKFTKLILRKRWQEFLQIRRQQRDVLLPLIRARRKIVEERKRSEQEDKKDYVQSYVDTLLDLELPEENRKLNEEDIMNLC 
M8a6n8                          GRRRCYVDTLLELRLGDEEMVSLCWEFMNAAAKITSTALEWIMARLVLHQARGVLRRHLSAAAPPPPPPPPRRYLAIPRRETKKDPPHALSPHFPRVSLA 
M7zhu4                          CAPSLLVLEPTASALQWIMANLVKRPDIQEAVRREIDAVVNADADEVSENVLGELEYLHAVVMKTLRLHPPTSFAFRQVMEEDHVVHNGQRIPVGTKVYF 
M8axb5                          RFLAFRGKQAELFLPLVNARRNTNHHHLKLSGGLPAYLDHLLDARVPVDDDTADGKALRPLTDDELVSLLSEFLGAGPGTVVSSMEWTLAHLVLQPEVQS 
M7z1d9                          LLAIHGRLGELYLPLVDARRESRPTCDGGGRRPYVDSLIDLRVPDGGKGDGAGRRAVRDDEFVNLLSEFLGAGTGTVMASIEWTLAHLVNDQEIQKKLRD 
M8CS19|M8CS19_AEGTA Cytochrome  LPAVARLIHRQRWSKLVALRRKQEEMYLPLIHARRSRQRKSGETPAYVDTLIDLRVPDDHNKRRRPRRLTDGELVGMCSEFLGAGTETVAAALQWIMANL 
M8A0V3|M8A0V3_TRIUA Cytochrome  ECHVRAVCSQVHGLQVAIGEVNPFSGPSLLAKLAEWRYQRQLLAFHARITELCLPLIAARR-RGPHDDDLCRPYVDILIQLRVPDGK-H--------GGR 
M8A8M2|M8A8M2_TRIUA Cytochrome  EALDEMQQMQLQISDGISRFPVLS-IFQPITKRLLRKEWALNVALRERQKGLMLPLIHA------------RRG-HDGAQCYADSLLELRVA----EEGG 
M7ZAM7|M7ZAM7_TRIUA Cytochrome  ATVRAIAAAQRELLIYRSRKMAIFSFLPSVTKHLYRDRLQTVHAMQRRKKDLFVPLINARREYRK--LGGEPKKESAFEHSYVDALLDIKLPE----EGN 
Lolium_Gaines_89A2              EVIDELQEMNRQIYLAMARFPAFC--FFPALS--MRRRWAQYKTLRERQSKVLLPLIHS------------SRG----TPCYAGSLLELRVP----EEGG 
A2_TRINITY_DN68910_c0_g1_i3|m.  -------------------------GFSMLARLTEWRYQGRLLAFYARITQLCLAIIAA-RRRRLRHDDICRPYVDALINLRVPQAEGS-----KDDGVR 
A2_TRINITY_DN66356_c0_g2_i1|m.  ERHLPALCSQVRGLLDAIGVVKPFAGTSLLARLAERRYQGRLLAFHARFTQLCLPI-------------------------------------------- 
A2_TRINITY_DN68910_c0_g2_i1|m.  ---CASLSSQHGGNGYAA------ATTSVVHMLMHSSTSA-----------------SPRKARMVAG------------ALLVTTRS------------- 
A2_TRINITY_DN70484_c0_g3_i1|m.  ---------------------------------------------------------------------------------------------------- 
A2_TRINITY_DN73066_c0_g3_i1|m.  EALDELQQMQLQISDAVSRFPVLS-IFQVITKRVLQKQWAGHVALRERQKELMLPLIRA------------PRGGHDGPLCYADSLLEQRVA-------- 
A2_TRINITY_DN77876_c5_g6_i1|m.  ---------------------------------VLQKQWAGHVALRERQKELMLPLIRA------------PRGGHEGPLCYADSLLEQRVA----EEGG 
A2_TRINITY_DN88769_c1_g2_i1|m.  ---------------------AVFSFLPAVTKHLFRDRLQNVQAMQRRKKELFVPLINARREYKSR--GGEPRRDTRFEHSYVDTLLDIKLP----EEGD 
A2_TRINITY_DN88769_c2_g3_i3|m.  AAVRAIATAQRDLLIYSSKKMGVFAFLPSLTKHLFRGRLQTVLAMRRRKMELFVPLINARREYKARAGGGEPRKETTFEHSYVDTLLDIKLPD---EEGN 
A2_TRINITY_DN88769_c2_g6_i2|m.  ---------------YSSKKMGVFAFLPSLTKHLFRGRLQTVLAMRRRKMELFVPLINARREYKTGAGAGEPRKETTFEHSYVDTLLDIKLPE---QEGN 
A2_TRINITY_DN91187_c2_g16_i2|m  ---------------------------------------------------------------------------------------------------- 
A3_TRINITY_DN101639_c0_g4_i1|m  EALEEMQQILLQISDPVSRFPMFEGIFLYDTA-------------------------------------------------------------------- 
A3_TRINITY_DN101639_c0_g5_i1|m  EALDELQQMQLQISDAVSRFPVLS-IFQVITKRVLQKQWAGHVALRERQKELMLPLIRA------------PRGGHEGPLCYADSLLEQRVA----EEGG 
A3_TRINITY_DN101639_c0_g6_i1|m  EALDELQQMQLQISDAVSRFPVLS-IFQVITKRVLQKQWAGHVALRERQKELMLPLIRA------------PRGGHDGPLCYADSLLEQRVA----EE-- 
A3_TRINITY_DN113586_c9_g10_i1|  ----------------------------------------------------------A------------PRAGEGGPRCYADSLVELRVP----EEGD 
A3_TRINITY_DN35959_c0_g1_i1|m.  ---------------MFEDTNQDESMLAAGEWKEKRTRMGKGLSAGSHSWTRPIPFHCG------------RRGCSWGSGHHVLNSLEREQPWIKGLGDG 
A3_TRINITY_DN47294_c0_g2_i1|m.  ------------------------------------------------------------NGVVLAAESIRTAMFGLQSEMAFGEGVDEGLIRVMSEAIS 
A3_TRINITY_DN70793_c0_g2_i1|m.  ----------------------------------------------------------------------------------------IKLPE----EGD 
A3_TRINITY_DN80133_c0_g1_i1|m.  ----AIATAQRDPLIYRSRKMAVFSFLPAVTKHLFRDRLQNVQAMQRRKKELFVPLINARREYKSR--GGEPRRDTRFEHSYVDTLLDIKLP----EEGD 
A3_TRINITY_DN93016_c2_g1_i2|m.  EDRVRALCSQVHGLQVGIGEFKPIAGFSMLARLTEWRYQGRLLAFYARITQLCLAIIAARRRR-LRHDDICRPYVDALINLRVPQAEGS-----KDDGVR 
A3_TRINITY_DN93016_c1_g2_i1|m.  ERRLPALCSQVQGLLDAIGVVKPFAGTSLLARLAERRYQDRLLAFHARFTQLCLPIIAARRGRLVRGDGLCRPYIDALIDLRVPQQAEEGGKDVDDGGRR 
A4_TRINITY_DN105332_c2_g3_i2|m  ---------------------------------------------------------------------------------------------------- 
A4_TRINITY_DN39512_c0_g1_i1|m.  ------MRDLIQAFVQSVDGATDFAGSKMAK-LLHWRRWRQFLAFRGRQAELFLPVINARRNADRHLRPNDGGLP-TYLDLLLDTRVPADDTADG--KAL 
A4_TRINITY_DN42484_c0_g1_i1|m.  ------------------------------------------------------------------------------------------D--EAVAAGG 
A4_TRINITY_DN76031_c0_g1_i1|m.  ---------------------------------------------------------------------------------------------------- 
A4_TRINITY_DN76068_c0_g3_i1|m.  SAVRAIATAQRDPLIYRSTKMAIFSFLPAVTKHLFRDRLQNVQAMQRRKKELFVPLINARREYKSR--GGQPRRDTRFEHSYVDTLLDIKLP----EEGD 
A4_TRINITY_DN77267_c1_g3_i1|m.  ---------------------------------------------------------------------------------------------------- 
A4_TRINITY_DN86750_c1_g4_i1|m.  ---------------------------------------------------------------------------------------------------- 
A4_TRINITY_DN86750_c1_g5_i1|m.  ---------------------------------------------------------------------------------------------------- 
A4_TRINITY_DN88254_c3_g1_i1|m.  EDRVRALCSQVHGLQVAIGEFKPIAGFSMLVRLTEWRYQGRLLAFYARITQLCLAIIAARRRR-LRHDDICRPYVDALINLRVPQAEGS-----KDDGVR 
A6_TRINITY_DN58600_c1_g3_i1|m.  NKMACMLMEEEADIADEEDETLAVFACLAK--LVALEEANADPKLGCSKVGR----KKSKP----------RMKLEGHAIFFSDYLVGN----------- 
A6_TRINITY_DN66770_c1_g1_i1|m.  -----------------------------------------------------------------------------FEHSYVDTLLDIKLPD---EEGN 
A7_TRINITY_DN126267_c0_g1_i1|m  -RHMRALCSQVYGFQVAIGAVKPFAASSMLAKLAEWRYQAGLLAFHARITDLCLPLIAARRQRLRGGDDLCRPYVDALIDLRVPQESKD--------GSR 
A7_TRINITY_DN163078_c0_g1_i1|m  ---------QISTMEIVLLGFLLSLVLCISAVGAVFRRRAWASPVHDEPQPIIEIRDPS------------IARQALMDNADSFCNRPLNIFPVALVSGP 
A7_TRINITY_DN73089_c0_g1_i1|m.  --RGVGRHVQMYIMEFVLLVFPLFLAFCITAV-ALLRRHGWAPAVHDGQQRIIEIRDPT------------IARRALMDNAEALCNRPLNLFPVALVSGR 
A7_TRINITY_DN73089_c1_g3_i1|m.  ----------LQAFVLSVDGAKDFAGSKTAK-LLHWRRWRRFLAFRGRQADLFLPLINARRNANRHVKRASGGMP-AYVDLLLDVRVPEEDGAGGDKALL 
A7_TRINITY_DN87364_c0_g1_i1|m.  ERHLPALCSQVRGLLDAIGVVKPFAGTSLLARLAERRYQDRLLAFHARFTQLCLPIIAARRRRLVRGDDLCRPYIDALIDLRVPQQAEEGGKDVDDGGRR 
A7_TRINITY_DN88932_c0_g2_i1|m.  --CELSLRFTVETMEVLVL---IGAVLCLVAV--LLRRSAMR---HRAQTTLHQIADTA------------AAHRALNEKASAFSNRPAAIFPVVLATGL 
A7_TRINITY_DN94510_c4_g1_i2|m.  EALEEMQQILLQISDAVSRFPMFEGIFLYDTANKLLRRRW--AALRERQMKLMLPLIRA------------PRGGHEGPLCYADSLLEQRVA----EEGG 
A7_TRINITY_DN94510_c4_g2_i1|m.  EALDELQQMQLQISDAVSRFPVLS-IFQVITKRVLQKQWAGHVALRERQKELMLPLIRA------------PRGGHEGPLCYADSLLEQRVA----EEGG 
A7_TRINITY_DN94953_c0_g1_i1|m.  AVLSRRRFHNMNSAPYGPLWRSMRRNLTSDIFHPLHLHRYASARRRALSGLLEDLRQQRPNGVVLAAESIRTAMFGLQAEMAFGEGVEEGLIRVMSEAIS 
A5_TRINITY_DN102218_c5_g6_i2|m  ---------------------HPPTSFVFRQVMDEDQAVHDGQRLPAGTKVFFTLAALARDGTVWDDPDEFKPQRFLAKE---GSGSDDNVVKMMPFGAG 
A5_TRINITY_DN103684_c0_g2_i1|m  EALDELQQMQLQISDAVSRFPVLS-IFQVITKRVLQKQWAGHVALRERQKELMLPLIRA------------PRGG------------------------- 
A5_TRINITY_DN103684_c0_g5_i1|m  ---------------AVSRFPVLS-IFQVITKRVLQKQWAGHVALRERQKELMLPLIRA------------PRGGHDGPLCYADSLLEQRVA----EEGG 
A5_TRINITY_DN103684_c1_g3_i3|m  ---------------------------------------------------------------------------------------------------- 
A5_TRINITY_DN34162_c0_g1_i1|m.  SFLFPDLPTPRAKKSVIPLATMPMSAPLRRPPLFRRRKSEAFDSLHLFSSSRALMAKEGSL----------RSQQSPRPARCSDTMDSDD---------- 
A5_TRINITY_DN81309_c2_g1_i1|m.  ---------------------------------------------------------------------------------------------------- 
A5_TRINITY_DN81309_c2_g2_i2|m.  ERRLPALCSQVQGLLDAIGVVKPFAGTSLLARLAERRYQGRLLAFHARFTQLCLPIIAARRGRLVRGDGLCRPYIDALIDLRVPQQAEEGGEDVDGGGRR 
A5_TRINITY_DN88739_c0_g1_i1|m.  EALEEMQQILLQISDAVSRFPMFEGIFLYDTANKLLRRRW--AALRERQMKLMLPLIRA------------PRGAEGGPRCYADSLVELRVP----EEGD 
A5_TRINITY_DN89190_c1_g7_i1|m.  ---------------------------------------------------------------------------------------------------- 
A5_TRINITY_DN93741_c1_g1_i1|m.  ---------------------------------------------------------------------------------------------------- 
A5_TRINITY_DN97327_c1_g3_i2|m.  --------AQRDLLIYSSKKMGVFAFLPSLTKHLFRGRLQTVLAMRRRKMELFVPLINARREYKTGAGAGEPRKETTFEHSYVDTLLDIKLPE---QEGN 
A8_TRINITY_DN28752_c0_g1_i1|m.  --HVRAMRDLLQAFVLSVDGAKDFAGSKTAK-LLHWRRWRRFLAFRGRQADLFLPLINARRNANRHVKRASGGMP-AYVDLLLDVRVPEEDGAGGDKALL 
A8_TRINITY_DN33033_c0_g1_i1|m.  NKMACMLMEEEADIADEEDETLAVFACLAK--LVALEEANADPKLGGSKVGR----RKTKP----------RMRLEGHAMLYADYFAENPRFDPKDFRRR 
A8_TRINITY_DN40046_c1_g1_i1|m.  ---------------------MPMSAPLRRPPLFRRRKSEAFDSLHLFSSSRALMAKEGSL----------RSQQSPRPARCSDTMDSDD---------- 
A8_TRINITY_DN42043_c0_g1_i1|m.  ERHLPALCSQVRGLLDAIGVVKPFAGTSLLARLAERRYQDRLLAFHARFTQLCLPIIAARRRRLVRGDDLCRPYIDALIDLRVPQQAEEGGKDVDDGGRR 
A8_TRINITY_DN49523_c2_g1_i1|m.  -----------------------------------------------------------------------------FEHSYVDTLLDIKLPD---EEGN 
A8_TRINITY_DN52955_c1_g4_i1|m.  EALDELQQMQLQISDAVSRFPVLS-IFQVITKRVLQKQWAGHVALRERQKELMLPLIRA------------PRGGHEGPLCYADSLLEQRVA----EEGG 
A8_TRINITY_DN52955_c1_g5_i1|m.  EALEEMQQILLQISDAVSRFPMFEGIFLYDTA--NKLLRRRWAALRERQMKLMLPLIRA------------PRGGAGGPRCYADSLVELRVP----EEGD 
A8_TRINITY_DN52955_c1_g6_i1|m.  QGDVRAAQRVMQEFVLGVGQATAYAGSKLAK-LLHWRRRRRFLASGGNMAAVFFPIIAAGQ-RRRASRPCMDGVF-PYVDSLLELRVPDND--ASILSDG 

                                        310       320       330       340       350       360       370       380       390       400         
                                ....|....|....|....|....|....|....|....|....|....|....|....|....|....|....|....|....|....|....|....|
Arapidopsis_gi|15217771|ref|NP  SEFLTAGTDTTATALQWIMANLVKYPEIQERLHEEIKSVVGEEAKEVEEEDVEKMPYLKAVVLEGLRRHPPGHFLLPHSVTEDTVLGGYKVPKNGTINFM 
M8a6n8                          APAPADKSAHDARYVFGALPAPAAMDSDDEKVLTALLEEEADAATQDEEHLMVPTALGGLLAGDAKPRRGGSAPGRRKAKNRHQVEGYCMLYSDYFADAP 
M7zhu4                          PLAAIARDSTAWDNPDEFKPQRFLASKGASEGTDNTIKMMPFGGGRRICPGRGVAMLHLSYFTANLVREFRWREGEGELAVDLQLHVEFFTVMKRPLCAH 
M8axb5                          NIRREVDAVEGALSEARVRQMKYMHAVVLESLRLHPPIPDMAHGKLYQAPPGTYSTNIKELGLCTVCREPCGRHPANNTQVARPFAADGPETLMKVTRQS 
M7z1d9                          EVDGAGGAVAASSSRSLIRGMPYLNAVVLETLRLHPQVPFVQRHVHADAAEVLGVRGKTSGDFIAQFTVGDMGRDGKTWIDPDEFRPERFLPGGEAEDVG 
M8CS19|M8CS19_AEGTA Cytochrome  VKRPHMQEAVRREINAAVDADAEEVGEEVLGKLEYLNAVLMEVLRLYPTATLVFRQVSEKDDIIHDGQRILTGTNVLFALKSLAQDKATWADPDEFKPER 
M8A0V3|M8A0V3_TRIUA Cytochrome  RALSDDEIVDLVLEFLGAGSGSLVVCLEWTLAHLVVQPEVQKKLRHEVDG--EAADR----SHLIRGMPYLHAVILESLRMHPPAPVALR---HGDMGRD 
M8A8M2|M8A8M2_TRIUA Cytochrome  RPLTDDEMVSLCSEFMIASTDTSVALLEWIMADLVNHPDVQAKLYEEVRG-KPELSEEELSG-----MPYLKAIVLEALRLHPTAHLVFP-----HGVQS 
M7ZAM7|M7ZAM7_TRIUA Cytochrome  RPLTDDEMINLCSEFLDAGTDTTSTGLQWIMAELVKNPAIQEKLYEEISA-TKGDDQDEVSEEDVHKMPYLKAVVLEGLRKHPPAHFVLP-----HKAAA 
Lolium_Gaines_89A2              RPLTDAEMVX------------------------------------------------------------------------------------------ 
A2_TRINITY_DN68910_c0_g1_i3|m.  RDLSDEEIVVLVVEFLGAGHASQVVCLEWTLAHLVAQPEVQEKVR----QELEDRETPDRSKKQICGRPYLQAVILESLRMHPPAPMAFR---HVQADAA 
A2_TRINITY_DN66356_c0_g2_i1|m.  ---------------------------------------------------------------------------------------------------- 
A2_TRINITY_DN68910_c0_g2_i1|m.  ---------YHVVEFLGAATGGPVACLEWTLAHLVAQPEVQEKLRREVDG--EAAISPDNMSQLIRSMPYLHAVLLESLRMH------------------ 
A2_TRINITY_DN70484_c0_g3_i1|m.  ---------------------------------------------------------------------------------------------------- 
A2_TRINITY_DN73066_c0_g3_i1|m.  ---------------------------------------------------------------------------------------------------- 
A2_TRINITY_DN77876_c5_g6_i1|m.  RPLTDDEMVSICSEFMMASTDTTVALLEWIMADLVKHPDVQAKLYEEVRR-KPELSDDDLRGG---GLPYLKAVVLEGLRLHPVAHVVFP-----HGGQS 
A2_TRINITY_DN88769_c1_g2_i1|m.  RPLTNDEMVNLCSEFLDAGTDTTSTGLQWIM--------------------------------------------------------------------- 
A2_TRINITY_DN88769_c2_g3_i3|m.  RPLTDIEMVNLCSEFLDAGTDTTSTGLQWIMAELVKNPSIQHKLYEEIKD-------------------------------------------------- 
A2_TRINITY_DN88769_c2_g6_i2|m.  RPLTDIEMVNLCSEFLDAGTDTTSTGLQWIMAELVKNPSIQHKLYEEIKD-TTGDDQEEISGEDVHKMPYLKAVVLEGLRKHPPGHFVLP-----HTAAE 
A2_TRINITY_DN91187_c2_g16_i2|m  -------------------MDTSLALLEWIMADLVNRPDVQAKLYEEVRG-KPDLREEDLSG-----MPYLKAIVLEGLRLHPTAHLVFP-----HGVQS 
A3_TRINITY_DN101639_c0_g4_i1|m  ---------------------------------------------------------------------------------------------------- 
A3_TRINITY_DN101639_c0_g5_i1|m  RPLTDDEMVSICSEFMMASTDTTVALLEWIMADLVNHPDAQAKLYEEVRG-KPD---------------------------------------------- 
A3_TRINITY_DN101639_c0_g6_i1|m  ---------------------------------------------------------------------------------------------------- 
A3_TRINITY_DN113586_c9_g10_i1|  RPLTDGEMVSLCTEFMIASMDTSLALLEWIMADLVKHPYVQAKLYEEVRG-KPDHREEDLSG-----MPYLKAIVLEGLRLHPTAHLVFP-----HGVQS 
A3_TRINITY_DN35959_c0_g1_i1|m.  LVCFNSKTKGITLPAHTIVALHGMRDKKHYLVILVYLFMKTATKSTKI---------------------------------------------------- 
A3_TRINITY_DN47294_c0_g2_i1|m.  DLVQSFPGLRILSLTKLPAFVAKVYYRERWNKLVTLRRNQEQMFLPLIRARRNGRRSHPGEAPAYVDTLTDLQVEGDGR--------------------- 
A3_TRINITY_DN70793_c0_g2_i1|m.  RPLTNDEMVNLCSEFLDAGTDTTSTGLQWIMAELVKNPSIQETLYKEISA-TTGDDQEEVSEEDVHKMPYLKAVVLEGLRKHPPAHFVLP-----HTA-- 
A3_TRINITY_DN80133_c0_g1_i1|m.  RPLTNDEMVNLCSEFLDAGTDTTSTGLQW----------------------------------------------------------------------- 
A3_TRINITY_DN93016_c2_g1_i2|m.  RDLSDEEIVVLVVEFLGAGHASQVVCLEWTLAHLVAQPEVQEKVRQELE----DRETPDRSKKQICGRPYLQAVILESLRMHPPAPLAFR---HVQANAA 
A3_TRINITY_DN93016_c1_g2_i1|m.  RALSDEE--------------------------------------------------------------------------------------------- 
A4_TRINITY_DN105332_c2_g3_i2|m  ---------------------------------------------------------------------------------------------------- 
A4_TRINITY_DN39512_c0_g1_i1|m.  RPLTDHELVSLVSEFLGAGPGTVVSSTEWTLAHLVLQPEAQNKIR------------------------------------------------------- 
A4_TRINITY_DN42484_c0_g1_i1|m.  ------------------------------------------------GGEAAE-ALSEKAIRD---MPYLRAVVLESVRIHPPSPFVTRGVHD-VATGR 
A4_TRINITY_DN76031_c0_g1_i1|m.  ----------------------------------------------------------------------------------PPAPMAFR---HVQADAA 
A4_TRINITY_DN76068_c0_g3_i1|m.  RPLTNDEMV------------------------------------------------------------------------------------------- 
A4_TRINITY_DN77267_c1_g3_i1|m.  ---------------------------------------------------------------------------------------------------- 
A4_TRINITY_DN86750_c1_g4_i1|m.  -------------------------------------------------G-KPDLREEDLSG-----MPYLKAIVLEGLRLHPTAHLVFP-----HGVQS 
A4_TRINITY_DN86750_c1_g5_i1|m.  -----------------------------------------------------------LSG-----MPYLKAIVLEGLRLHPTAHLVFP-----HGVQS 
A4_TRINITY_DN88254_c3_g1_i1|m.  RDLSDEEIVVLVVEFLGAGHASQVVCLEWTLAHLVAQPEVQEKVRQELE----DRETPDRSKKQICGRPYLQAVILESLRMHPPAPLAFR---HVQANAA 
A6_TRINITY_DN58600_c1_g3_i1|m.  --------LKFMQRISVED--FRWAG-----LCFSSWCNMCGSM---AHGSR------------------------------------------------ 
A6_TRINITY_DN66770_c1_g1_i1|m.  RPLTDIEMVNLCSEFLDAGTDTTSTGLQWIMAELVKNPSIQHKLYEEIKD-TTGDDQEEISGEDVHKMPYLKAVVLEGLRKHPPGHFVLP-----HTAAE 
A7_TRINITY_DN126267_c0_g1_i1|m  RALSDDEIVDHVVEFLGAATGGPVACLEWTLAHLVAQPEVQEKLRREVDG--EAAISPDNMSQLIRSMPYLHAVLLESLRMHPPAPVALR---HVQAN-- 
A7_TRINITY_DN163078_c0_g1_i1|m  RRRLSDNLTSVPYGPRWRALRCTLNAAVLHPSRLGHMDPLRLVAMDALVVDLRDRVRRG-GGGEIVVRDILNAAVFPLVARTCF---------------- 
A7_TRINITY_DN73089_c0_g1_i1|m.  RRRLSDNITSVPYGPRWRALRCTLNAAVLHPTRLGHMDPLRLDAMDSLVADLRGRVRRGHGGGEIVVRDILNAAVFPMVARTCFGSGVDEGHVRAMRDLL 
A7_TRINITY_DN73089_c1_g3_i1|m.  RPLTDDGLVSLLSEFLGAGPGTMVSSMEWTLAHLVLQTEVQKKIRH--EVDAVEGALSEAHVRQ---MKYMRAVVLESFRLHPPIPLLLRDVPDQVSAAA 
A7_TRINITY_DN87364_c0_g1_i1|m.  RALSDEEMVSLMLEFLGAGMMSQVACLEWTLAHLVADPEVQEKLRREVVEAAVDGEAPDSIQQLTRGMPYLHAVLLESLRLHPPAPMAFR---HVQADDA 
A7_TRINITY_DN88932_c0_g2_i1|m.  RGERNENITTVDYGPHWRALRCNLT--------------------------------------------------------------------------- 
A7_TRINITY_DN94510_c4_g1_i2|m.  RPLTDDEMVSICSEFMMASTDTTVALLEWIMADLVKHPDVQAKLYEEVRR-KPELSDDDLRGG---GLPYLKAVVLEGLRLHPVAHVVFP-----HGGQS 
A7_TRINITY_DN94510_c4_g2_i1|m.  RPLTDDEMVSICSEFMMASTDTTVALLEWIMADLVNHPDAQAKLYEEVRG-KPDLREEDLSG-----MPYLKAIVLEGLRLHPTAHLVFP-----HGVQS 
A7_TRINITY_DN94953_c0_g1_i1|m.  DLVQSFPALRILSLTKLPAFVAKVYYRERWNKLVTLRRNQEQMFLPLIRARRNGRRSHPGEAPAYVDTLTDLQVEGDGRSLADGELVGMCSEFLGAGTET 
A5_TRINITY_DN102218_c5_g6_i2|m  RRMCPGKGIAMLRISYFAANLLREFEWSEPEGELPVDLEPKVEFFTVMKHPLRAHLEQLGRPRA------------------------------------ 
A5_TRINITY_DN103684_c0_g2_i1|m  ---------------------------------------------------------------------------------------------------- 
A5_TRINITY_DN103684_c0_g5_i1|m  RPLTDDEMVSICSEFMMASTDTTVALLEWIMAD------------------------------------------------------------------- 
A5_TRINITY_DN103684_c1_g3_i3|m  --------VSLCTEFMIASMDTSLALLEWIMADLVKHPDVQAKLYEEVRG-KPELSDDDLRGG---GLPYLKAVVLEGLRLHPVAHVVFP-----HGGQS 
A5_TRINITY_DN34162_c0_g1_i1|m.  --------EEMLVELLAEEVDAGLDDDKEHLLMLAALGGLLVEN----SKSR------------------------------------------------ 
A5_TRINITY_DN81309_c2_g1_i1|m.  ---------------------------------------------------------------------------------------------------- 
A5_TRINITY_DN81309_c2_g2_i2|m.  -ALSDEEMVSLMLEFLGAGMMSQVACLEWTLAHLVADPEAQEKLRREVVEAAVDGEGPDSIQQLTRGMPYLHAVLLESLRMHPPAPLAFR---HVQANAA 
A5_TRINITY_DN88739_c0_g1_i1|m.  RPLTDGEMVSLCTEFMIASMDTSLALLEWIMADLVKHPDVQAKLYEEVRR-KPELSDDDL---------------------------------------- 
A5_TRINITY_DN89190_c1_g7_i1|m.  -------------------------------------------------G-KPDLREEDLSG-----MPYLKAIVLEGLRLHPTAHLVFP-----HGVQS 
A5_TRINITY_DN93741_c1_g1_i1|m.  -----------------------------------------------------------------------------SLRMHPPAPMAFR---HVQADAA 
A5_TRINITY_DN97327_c1_g3_i2|m.  RPLTDIEMVNLCSEFLDAGTDTTSTGLQWIMAELVKNPSIQHKLYEEIKD-TTGDDQEEISGEDVHKMPYLKAVVLEGLRKHPPGHFVLP-----HTAAE 
A8_TRINITY_DN28752_c0_g1_i1|m.  RPLTDDELVSLLSEFLGAGPGTMVSSMEWTLAHLVLQTEVQKKIRH--EVDAVEGALSEAHVRQ---MKYMRAVVLESFRLHPPIPLLLRDVPDQVSAAA 
A8_TRINITY_DN33033_c0_g1_i1|m.  FRMGRPLFLKLVQHVREYDPWFKMKKDAVGMLGFSSLQKCTAAMRMLAYGAPADGQVDYLRMSESTA--------------------------------- 
A8_TRINITY_DN40046_c1_g1_i1|m.  --------EEMLVELLAEEVDAGLDDDKEHLLMLAALGGLLVEN----SKSR------------------------------------------------ 
A8_TRINITY_DN42043_c0_g1_i1|m.  RALSDEEMVSLMLEFLGAGMMSQVACLEWTLAHLVADPEVQEKLRREVVEAAVDGEAPDSIQQLTRGMPYLHAVLLESLRLHPPAPMAFR---HVQADDA 
A8_TRINITY_DN49523_c2_g1_i1|m.  RPLTDIEMVNLCSEFLDAGTDTTSTGLQWIMAELVKNPSIQHKLYEEIKD-TTGDDQEEISGEDVHKMPYLKAVVLEGLRKHPPGHFVLP-----HTAAE 
A8_TRINITY_DN52955_c1_g4_i1|m.  RPLTDDEMVSICSEFMMASTDTTVALLEWIMADLVNHPDAQAKLYEEVRG-KPDLREEDLSGM---PY--LKAIVLEGLRLHPTAHLVFP-----HGVQS 
A8_TRINITY_DN52955_c1_g5_i1|m.  RPLTDGEMVSLCTEFMIASMDTSLALLEWIMADLVKHPDVQAKLYEEVRG-KPELSDDDLRGG---GLPYLKAVVLEGLRLHPVAHVVFP-----HGGQS 
A8_TRINITY_DN52955_c1_g6_i1|m.  RALTDDEMVSLVSEFLGAGTETVVSCVEFTLAHLVTQPEVQNRLRREIDGEAGKGVLSNDELRQRA-MPYLHAVVLESLRMHPTVPFVMRDVRP-EDAAA 

                                        410       420       430       440       450       460       470       480       490       500         
                                ....|....|....|....|....|....|....|....|....|....|....|....|....|....|....|....|....|....|....|....|
Arapidopsis_gi|15217771|ref|NP  VAEIGRDPVEWEEPMAFKPERFMGEEEAVDLTGSRGIKMMPFGAGRRICPGIGLAMLHLEYYVANMVREFQWKEVQGHEVDLTEKLEFTVVMKHPLKALA 
M8a6n8                          LHGDKVFRRHYRMGRKLFLRIINELKEYDNYFRCKKDCTGTLGFTSIQKCMTAMRMLAYGAPGEVDGNCTADGERRSPFLDAVVLEALRRHPPAHYLLAH 
M7zhu4                          LELRGIRDQN                                                                                           
M8axb5                          MRRIRLGDDETGRLLDILPPNNICIGVPFVTGLMNTNLMTKHVMWPSRLGCWSSKNEGRTLPIRIGVLREKFKPASGEAEIWFWRPFLQKLRR        
M7z1d9                          PLPGTKEIRMMPFGAGHRFCPGVGLAMMNIKCFLAALVHEFEWAPPGTEGCAGVDMTELNTFIKAMKKPLSARLTRRT                       
M8CS19|M8CS19_AEGTA Cytochrome  FLADKGGENVNLVAAAGSGGEIRMIPFGAGRRVCPGMGIAMLHMGYFTANLVREFEWREAEGELAVNLRPHFGFFTVMKHPLRAHLAVLPRREGISKGGV 
M8A0V3|M8A0V3_TRIUA Cytochrome  -------------------------MKAWKDTDEFRPERFLAGGDAEGVGPLPGRKETRMMPFGAGHRFCPGVGLAMATMKGLFELKDFHRNIGGFQSIG 
M8A8M2|M8A8M2_TRIUA Cytochrome  DTEIGGYMVPKGVDINFLVSDFGLDETVWPAAREFRPERFLHDHG---VDITCTKEIK-MAPFGAGRRMCPGYVLATLHAEYFVGSLVREFQWLPPGGQD 
M7ZAM7|M7ZAM7_TRIUA Cytochrome  DMEIGGYLIPKGATVNFMVAEMSRDEREWEKPTEFVPERFLPGGAGEAVDVTGNREIK-MMPFGVGRRICAGLGIAMLHLEYFVANMVREFEWQEVAGEE 
Lolium_Gaines_89A2              ---------------------------------------------------------------------------------------------------- 
A2_TRINITY_DN68910_c0_g1_i3|m.  STVMGEGGAESDVVVLFLLGDMGRDAKAWKDPDVFRPERFLAGGDAEGVGPLPGGKETRMLPFGAGHRHCPGMGFAMANIKCFLAALVREFEWAPPTTNA 
A2_TRINITY_DN66356_c0_g2_i1|m.  ---------------------------------------------------------------------------------------------------- 
A2_TRINITY_DN68910_c0_g2_i1|m.  ---------------------------------------------------------------------------------------------------- 
A2_TRINITY_DN70484_c0_g3_i1|m.  ---------------------------------------------------------------------------------------------------- 
A2_TRINITY_DN73066_c0_g3_i1|m.  ---------------------------------------------------------------------------------------------------- 
A2_TRINITY_DN77876_c5_g6_i1|m.  DAEVGGYMVPTGALINFLVGDFGLDETVWPAAREYRPERFMHDHD---VDITGSKEIK-MAPFSAGRRMCPGYVLALLHAEYLVGSLVREFQWL------ 
A2_TRINITY_DN88769_c1_g2_i1|m.  ---------------------------------------------------------------------------------------------------- 
A2_TRINITY_DN88769_c2_g3_i3|m.  ---------------------------------------------------------------------------------------------------- 
A2_TRINITY_DN88769_c2_g6_i2|m.  DMELGGYLIPKGSTVNFMVAEMGRDEREWEKPTEFVPERFLPGGVGEGIDVTGNREIK-MMPFGVGRRICAGLGIAMLHLEY------------------ 
A2_TRINITY_DN91187_c2_g16_i2|m  DAELGGYVVPKGADINFLVADFGLDETVWPAAREFRPERFLDDHG---VDITCTKGIK-MAPFGAGRRMCPGYVLATLHLEYFVGSLVRDFQWLPP-AQD 
A3_TRINITY_DN101639_c0_g4_i1|m  ---------------------------------------------------------------------------------------------------- 
A3_TRINITY_DN101639_c0_g5_i1|m  ---------------------------------------------------------------------------------------------------- 
A3_TRINITY_DN101639_c0_g6_i1|m  ---------------------------------------------------------------------------------------------------- 
A3_TRINITY_DN113586_c9_g10_i1|  DAELGGYVVPKG---------------------------------------------------------------------------------------- 
A3_TRINITY_DN35959_c0_g1_i1|m.  ---------------------------------------------------------------------------------------------------- 
A3_TRINITY_DN47294_c0_g2_i1|m.  ---------------------------------------------------------------------------------------------------- 
A3_TRINITY_DN70793_c0_g2_i1|m.  ---------------------------------------------------------------------------------------------------- 
A3_TRINITY_DN80133_c0_g1_i1|m.  ---------------------------------------------------------------------------------------------------- 
A3_TRINITY_DN93016_c2_g1_i2|m.  STIVG--GVDTNMMVLLVLGDIGRDRKAWKDPDEFRPERFVTGGEAEGAGPLSGGKDTRMLQFGAGHRHSPGMSFAMANIKCFLAALVCGFEWA-PRRND 
A3_TRINITY_DN93016_c1_g2_i1|m.  ---------------------------------------------------------------------------------------------------- 
A4_TRINITY_DN105332_c2_g3_i2|m  ---------------------------------------------------------------------------------------------------- 
A4_TRINITY_DN39512_c0_g1_i1|m.  ---------------------------------------------------------------------------------------------------- 
A4_TRINITY_DN42484_c0_g1_i1|m.  LR----------KRLIFMVREIGRHGSAWTEPRKFRPERFLPGGEAEDVGHMPGRNEIRMMPFGGGRRFCPGSNL------------------------- 
A4_TRINITY_DN76031_c0_g1_i1|m.  STVMGEGGAESDVVVLFLLGDMGRDAKAWKDPDVFRPERFLAGGDAEGVGPLPGGKETRMLPFGAGHRHCPGMAFAMANIKCFLAALVREFEWAPPTTNA 
A4_TRINITY_DN76068_c0_g3_i1|m.  ---------------------------------------------------------------------------------------------------- 
A4_TRINITY_DN77267_c1_g3_i1|m.  VGCDATVVPKQGMRAHFNLGDIGRDKNSWTDPDAFCPERFLAGGEGEGVGLVPGPNEIRMMPFGAGRRACPGVGLATMHVKSFVAALVREFDWAPPARGG 
A4_TRINITY_DN86750_c1_g4_i1|m.  DAELGGYTVPKGADINFLVADFGLDETVWPAAREFRPDRFLDDHG---VDITCTKGIK-MAPFGAGRRMCP----------------------------- 
A4_TRINITY_DN86750_c1_g5_i1|m.  DADLGGYVVPKGADINFLVADFGLDETVWPAAREFRPERFLDDHG---VDITCTKGIK-MAPFGAGRRMCPGYLLATLHLEYFVGSLVRDFQWLPP-PAQ 
A4_TRINITY_DN88254_c3_g1_i1|m.  STIVG--GVDTNMMV------------------------------------------------------------------------------------- 
A6_TRINITY_DN58600_c1_g3_i1|m.  ---------------------------------------------------------------------------------------------------- 
A6_TRINITY_DN66770_c1_g1_i1|m.  DMELGGYLIPKGSTVNFMVAEMGRDEREWEKPTEFVPERFLPG--------------------------------------------------------- 
A7_TRINITY_DN126267_c0_g1_i1|m  -----------------------------------AAAKLVGG----GTTAVPADNEL------------------------------------------ 
A7_TRINITY_DN163078_c0_g1_i1|m  ---------------------------------------------------------------------------------------------------- 
A7_TRINITY_DN73089_c0_g1_i1|m.  Q--------------------------------------------------------------------------------------------------- 
A7_TRINITY_DN73089_c1_g3_i1|m.  VGCDATVVPKQGMRAHFNLGDIGRDKNS------------------------------------------------------------------------ 
A7_TRINITY_DN87364_c0_g1_i1|m.  STVMGEGGTDSDVVVLFLLGDMGRDAKAWKDPDVFRPERFLAGGDAEGVGPLPGGKETRMLPFGAGHRHCPGMGFAMANIKCFLAALVREFEWV-PPTNT 
A7_TRINITY_DN88932_c0_g2_i1|m.  ---------------------------------------------------------------------------------------------------- 
A7_TRINITY_DN94510_c4_g1_i2|m.  DAEVGGYMVPTGALINFLVGDFGLDETVWPAAREYRPERFMHDHG---VDITGSKEIK-MAPFSAGRRMCPGYVLALLHAEYLVGSLVREFQWLPPPQGV 
A7_TRINITY_DN94510_c4_g2_i1|m.  DAELGGYVVPKGADINFLVADFGLDEAVWPAAREFRPERFMDDHG---VDITCTKGIK-MAPFGAGRRMCPGYVLATLHLEYFVGSLVRDFQWLPPPAQD 
A7_TRINITY_DN94953_c0_g1_i1|m.  VAAELQWIVAELVKRPHMQEAIRREIDAVVGADAEEVSEDVLGKLEYLNAVIMEGLRLHPAVHTVFREVMERDHVFLDGRRIRTGTTVAFPVASPALDKT 
A5_TRINITY_DN102218_c5_g6_i2|m  ---------------------------------------------------------------------------------------------------- 
A5_TRINITY_DN103684_c0_g2_i1|m  ---------------------------------------------------------------------------------------------------- 
A5_TRINITY_DN103684_c0_g5_i1|m  ---------------------------------------------------------------------------------------------------- 
A5_TRINITY_DN103684_c1_g3_i3|m  DAEVGGYMVPTGALINFLVGDFGLDETVWPAAREYRPERFMHDHD---VDITGSKEIK-MAPFSAGRRMCPGYV-------------------------- 
A5_TRINITY_DN34162_c0_g1_i1|m.  ---------------------------------------------------------------------------------------------------- 
A5_TRINITY_DN81309_c2_g1_i1|m.  ---------------------------------------------------------------------------------------------------- 
A5_TRINITY_DN81309_c2_g2_i2|m.  STIVG--GVDTNMMVLLVLGDIGRDRKAWKDPDEFRPERFVTGGEAEGAGPLSGGKDTRMLQFGAGHRHSPGMSFAMANIKCFLAALVCGFEWA-PRR-N 
A5_TRINITY_DN88739_c0_g1_i1|m.  ---------------------------------------------------------------------------------------------------- 
A5_TRINITY_DN89190_c1_g7_i1|m.  DAELGGYVVPKGADINFLVADFGLDEAVWPAAREFRPERFMDDHG---VDITCTKGIK-MAPFGAGRRMCPGYVLATLHLEYFVGSLVR----------- 
A5_TRINITY_DN93741_c1_g1_i1|m.  STVMGEGGAESDVVVLFLLGDMGRDAKAWKDPDVFRPERFLAGGDAEGVGPLPGGKETRMLPFGAGHRHCPGMGFAMANIKFFLAALVREFEWAPPTTNA 
A5_TRINITY_DN97327_c1_g3_i2|m.  DMELGGYLIPKGSTVNFMVAEMGRDEREWEKPTEFVPERFLPGGVGEGIDVTGNREIK-MMPFGVGRRICAGLGIAMLHLEYF----------------- 
A8_TRINITY_DN28752_c0_g1_i1|m.  VGCDATVVPKQGMRAHFNLGDIGRDKNSWTDPDAFCPERFLAGGEGEGVGLVPGPKEIRMMPFGAGRRACPGVGLATMHVKCFVAALVREFDWAPPARGG 
A8_TRINITY_DN33033_c0_g1_i1|m.  ---------------------------------------------------------------------------------------------------- 
A8_TRINITY_DN40046_c1_g1_i1|m.  ---------------------------------------------------------------------------------------------------- 
A8_TRINITY_DN42043_c0_g1_i1|m.  STVMGEGGTDSDVVVLFLLGDMGRDAKAWKDPDVFRPERFLAGGDAEGVGPLPGGKETRMLPFGAGHRHCPGMGFAMANIKCFLAALVREFEWV-PPTNT 
A8_TRINITY_DN49523_c2_g1_i1|m.  DMELGGYLIPKGSTVNFMVAEMGRDEREWEKPTEFVPERFLPG--------------------------------------------------------- 
A8_TRINITY_DN52955_c1_g4_i1|m.  DAELGGYVVPKGADINFLVADFGLDEAVWPAAREFRPERFMDDHG---VDITCTKGIK-MAPFGAGRRMCPGYVLATLHLEYFVGSLVRDFQWLPPPAQD 
A8_TRINITY_DN52955_c1_g5_i1|m.  DAEVGGYMVPTGALINFLVGDFGLDETVWPAAREYRPERFMHDHG---VDITGSKEIK-MAPFSAGRRMCPGYVLALLHAEYLVGSLVREFQWLPPPQGV 
A8_TRINITY_DN52955_c1_g6_i1|m.  VAGEATVTA-AGLRVHFMLRDIGRDSKTWTDPDDFRPERFMPGGEAEDVGPLPGPKEIIMMPFGAGRRFCPGMGLAMLHAKLLLVALIREFEWAPLAAGQ 

                                        510       520       530       540       550       560       570       580       590       600         
                                ....|....|....|....|....|....|....|....|....|....|....|....|....|....|....|....|....|....|....|....|
Arapidopsis_gi|15217771|ref|NP  VPRRCH                                                                                               
M8a6n8                          TTDKDVYLDGYVIPKGSVVNYGVADIGRDATSWTNPVEFLPERFLEGGEGYGVSVTAGSGSGEESMKMMPFGSGRRACPGATVALTVLKSFVEKLVTRFE 
M7zhu4                                                                                                                               
M8axb5                                                                                                                               
M7z1d9                                                                                                                               
M8CS19|M8CS19_AEGTA Cytochrome  NSRKEE                                                                                               
M8A0V3|M8A0V3_TRIUA Cytochrome  FFPKEAFWIKGTTPPKFLWIHSYTSIL------------------------------------------------------------------------- 
M8A8M2|M8A8M2_TRIUA Cytochrome  TVDMTEELAMAINMKHPLRARIIPRA-------------------------------------------------------------------------- 
M7ZAM7|M7ZAM7_TRIUA Cytochrome  VDFAEKNEFTVVMKKPLRPRPVLKTTVSTRLLKRDLKIAQAASSGITPRGTSLQFITTSREGWRNRVKPHNNTKEDVIVITPIASQKFSLKDTRTTKI-- 
Lolium_Gaines_89A2              ---------------------------------------------------------------------------------------------------- 
A2_TRINITY_DN68910_c0_g1_i3|m.  AVVDFTELDGFLKTMKKPLAARLTRRTSASSV-------------------------------------------------------------------- 
A2_TRINITY_DN66356_c0_g2_i1|m.  ---------------------------------------------------------------------------------------------------- 
A2_TRINITY_DN68910_c0_g2_i1|m.  ---------------------------------------------------------------------------------------------------- 
A2_TRINITY_DN70484_c0_g3_i1|m.  ---------------------------------------------------------------------------------------------------- 
A2_TRINITY_DN73066_c0_g3_i1|m.  ---------------------------------------------------------------------------------------------------- 
A2_TRINITY_DN77876_c5_g6_i1|m.  ---------------------------------------------------------------------------------------------------- 
A2_TRINITY_DN88769_c1_g2_i1|m.  ---------------------------------------------------------------------------------------------------- 
A2_TRINITY_DN88769_c2_g3_i3|m.  ---------------------------------------------------------------------------------------------------- 
A2_TRINITY_DN88769_c2_g6_i2|m.  ---------------------------------------------------------------------------------------------------- 
A2_TRINITY_DN91187_c2_g16_i2|m  TVDMTEELAMAINMKHPLRARIIPRA-------------------------------------------------------------------------- 
A3_TRINITY_DN101639_c0_g4_i1|m  ---------------------------------------------------------------------------------------------------- 
A3_TRINITY_DN101639_c0_g5_i1|m  ---------------------------------------------------------------------------------------------------- 
A3_TRINITY_DN101639_c0_g6_i1|m  ---------------------------------------------------------------------------------------------------- 
A3_TRINITY_DN113586_c9_g10_i1|  ---------------------------------------------------------------------------------------------------- 
A3_TRINITY_DN35959_c0_g1_i1|m.  ---------------------------------------------------------------------------------------------------- 
A3_TRINITY_DN47294_c0_g2_i1|m.  ---------------------------------------------------------------------------------------------------- 
A3_TRINITY_DN70793_c0_g2_i1|m.  ---------------------------------------------------------------------------------------------------- 
A3_TRINITY_DN80133_c0_g1_i1|m.  ---------------------------------------------------------------------------------------------------- 
A3_TRINITY_DN93016_c2_g1_i2|m.  VVD-FTELDGFLKTMKKPLAARLTRRTPAASA-------------------------------------------------------------------- 
A3_TRINITY_DN93016_c1_g2_i1|m.  ---------------------------------------------------------------------------------------------------- 
A4_TRINITY_DN105332_c2_g3_i2|m  ---------------------------------------------------------------------------------------------------- 
A4_TRINITY_DN39512_c0_g1_i1|m.  ---------------------------------------------------------------------------------------------------- 
A4_TRINITY_DN42484_c0_g1_i1|m.  ---------------------------------------------------------------------------------------------------- 
A4_TRINITY_DN76031_c0_g1_i1|m.  AVVDFTELDGFLKTMKKPLAARLTRRTSASSV-------------------------------------------------------------------- 
A4_TRINITY_DN76068_c0_g3_i1|m.  ---------------------------------------------------------------------------------------------------- 
A4_TRINITY_DN77267_c1_g3_i1|m.  -VDLTEL--------------------------------------------------------------------------------------------- 
A4_TRINITY_DN86750_c1_g4_i1|m.  ---------------------------------------------------------------------------------------------------- 
A4_TRINITY_DN86750_c1_g5_i1|m.  D--------------------------------------------------------------------------------------------------- 
A4_TRINITY_DN88254_c3_g1_i1|m.  ---------------------------------------------------------------------------------------------------- 
A6_TRINITY_DN58600_c1_g3_i1|m.  ---------------------------------------------------------------------------------------------------- 
A6_TRINITY_DN66770_c1_g1_i1|m.  ---------------------------------------------------------------------------------------------------- 
A7_TRINITY_DN126267_c0_g1_i1|m  ---------------------------------------------------------------------------------------------------- 
A7_TRINITY_DN163078_c0_g1_i1|m  ---------------------------------------------------------------------------------------------------- 
A7_TRINITY_DN73089_c0_g1_i1|m.  ---------------------------------------------------------------------------------------------------- 
A7_TRINITY_DN73089_c1_g3_i1|m.  ---------------------------------------------------------------------------------------------------- 
A7_TRINITY_DN87364_c0_g1_i1|m.  AVVDFTELDGFLKTMNKPLAARLTRRTTASV--------------------------------------------------------------------- 
A7_TRINITY_DN88932_c0_g2_i1|m.  ---------------------------------------------------------------------------------------------------- 
A7_TRINITY_DN94510_c4_g1_i2|m.  DMTEELAMAINMKHPLRARIIPRA---------------------------------------------------------------------------- 
A7_TRINITY_DN94510_c4_g2_i1|m.  TVDMTEELAMAINMKHPLRARIIPRA-------------------------------------------------------------------------- 
A7_TRINITY_DN94953_c0_g1_i1|m.  AWADPKEFNPERFMATGGGEKTSLLAGAGSAGEIRMMPFGAGRRMCPGMGVAMLHMAYFLANILREFEWKDPEGELAVDLKPQWPRNEGFFNVMKRPLRA 
A5_TRINITY_DN102218_c5_g6_i2|m  ---------------------------------------------------------------------------------------------------- 
A5_TRINITY_DN103684_c0_g2_i1|m  ---------------------------------------------------------------------------------------------------- 
A5_TRINITY_DN103684_c0_g5_i1|m  ---------------------------------------------------------------------------------------------------- 
A5_TRINITY_DN103684_c1_g3_i3|m  ---------------------------------------------------------------------------------------------------- 
A5_TRINITY_DN34162_c0_g1_i1|m.  ---------------------------------------------------------------------------------------------------- 
A5_TRINITY_DN81309_c2_g1_i1|m.  ---------------------------------------------------------------------------------------------------- 
A5_TRINITY_DN81309_c2_g2_i2|m.  DVVDFTELDGFLKTMKKPLAARLTRRTPAASA-------------------------------------------------------------------- 
A5_TRINITY_DN88739_c0_g1_i1|m.  ---------------------------------------------------------------------------------------------------- 
A5_TRINITY_DN89190_c1_g7_i1|m.  ---------------------------------------------------------------------------------------------------- 
A5_TRINITY_DN93741_c1_g1_i1|m.  AVVDFTELDGFLKTMKKPLAARLTRRTSASSV-------------------------------------------------------------------- 
A5_TRINITY_DN97327_c1_g3_i2|m.  ---------------------------------------------------------------------------------------------------- 
A8_TRINITY_DN28752_c0_g1_i1|m.  -VDLTELDGFFKVMKTPLRARVTLRERDCV---------------------------------------------------------------------- 
A8_TRINITY_DN33033_c0_g1_i1|m.  ---------------------------------------------------------------------------------------------------- 
A8_TRINITY_DN40046_c1_g1_i1|m.  ---------------------------------------------------------------------------------------------------- 
A8_TRINITY_DN42043_c0_g1_i1|m.  AVVDFTELDGFLKTMNKPLAARLTRRTTASV--------------------------------------------------------------------- 
A8_TRINITY_DN49523_c2_g1_i1|m.  ---------------------------------------------------------------------------------------------------- 
A8_TRINITY_DN52955_c1_g4_i1|m.  TVDMTEELAVAINMKHPLRARIIPRA-------------------------------------------------------------------------- 
A8_TRINITY_DN52955_c1_g5_i1|m.  DMTEELAVAINMKHPLRARIIPRA---------------------------------------------------------------------------- 
A8_TRINITY_DN52955_c1_g6_i1|m.  KVDLTELDGFFKTMKKPLRARITQRLKAAP---------------------------------------------------------------------- 

                                        610       620       630        
                                ....|....|....|....|....|....|....|..
Arapidopsis_gi|15217771|ref|NP                                        
M8a6n8                          WTPVGAVDMEEKPGLVTEMRTPLRTCLVVRPHVQPTN 
M7zhu4                                                                
M8axb5                                                                
M7z1d9                                                                
M8CS19|M8CS19_AEGTA Cytochrome                                        
M8A0V3|M8A0V3_TRIUA Cytochrome  ------------                          
M8A8M2|M8A8M2_TRIUA Cytochrome  ------------                          
M7ZAM7|M7ZAM7_TRIUA Cytochrome  ------------                          
Lolium_Gaines_89A2              ------------                          
A2_TRINITY_DN68910_c0_g1_i3|m.  ------------                          
A2_TRINITY_DN66356_c0_g2_i1|m.  ------------                          
A2_TRINITY_DN68910_c0_g2_i1|m.  ------------                          
A2_TRINITY_DN70484_c0_g3_i1|m.  ------------                          
A2_TRINITY_DN73066_c0_g3_i1|m.  ------------                          
A2_TRINITY_DN77876_c5_g6_i1|m.  ------------                          
A2_TRINITY_DN88769_c1_g2_i1|m.  ------------                          
A2_TRINITY_DN88769_c2_g3_i3|m.  ------------                          
A2_TRINITY_DN88769_c2_g6_i2|m.  ------------                          
A2_TRINITY_DN91187_c2_g16_i2|m  ------------                          
A3_TRINITY_DN101639_c0_g4_i1|m  ------------                          
A3_TRINITY_DN101639_c0_g5_i1|m  ------------                          
A3_TRINITY_DN101639_c0_g6_i1|m  ------------                          
A3_TRINITY_DN113586_c9_g10_i1|  ------------                          
A3_TRINITY_DN35959_c0_g1_i1|m.  ------------                          
A3_TRINITY_DN47294_c0_g2_i1|m.  ------------                          
A3_TRINITY_DN70793_c0_g2_i1|m.  ------------                          
A3_TRINITY_DN80133_c0_g1_i1|m.  ------------                          
A3_TRINITY_DN93016_c2_g1_i2|m.  ------------                          
A3_TRINITY_DN93016_c1_g2_i1|m.  ------------                          
A4_TRINITY_DN105332_c2_g3_i2|m  ------------                          
A4_TRINITY_DN39512_c0_g1_i1|m.  ------------                          
A4_TRINITY_DN42484_c0_g1_i1|m.  ------------                          
A4_TRINITY_DN76031_c0_g1_i1|m.  ------------                          
A4_TRINITY_DN76068_c0_g3_i1|m.  ------------                          
A4_TRINITY_DN77267_c1_g3_i1|m.  ------------                          
A4_TRINITY_DN86750_c1_g4_i1|m.  ------------                          
A4_TRINITY_DN86750_c1_g5_i1|m.  ------------                          
A4_TRINITY_DN88254_c3_g1_i1|m.  ------------                          
A6_TRINITY_DN58600_c1_g3_i1|m.  ------------                          
A6_TRINITY_DN66770_c1_g1_i1|m.  ------------                          
A7_TRINITY_DN126267_c0_g1_i1|m  ------------                          
A7_TRINITY_DN163078_c0_g1_i1|m  ------------                          
A7_TRINITY_DN73089_c0_g1_i1|m.  ------------                          
A7_TRINITY_DN73089_c1_g3_i1|m.  ------------                          
A7_TRINITY_DN87364_c0_g1_i1|m.  ------------                          
A7_TRINITY_DN88932_c0_g2_i1|m.  ------------                          
A7_TRINITY_DN94510_c4_g1_i2|m.  ------------                          
A7_TRINITY_DN94510_c4_g2_i1|m.  ------------                          
A7_TRINITY_DN94953_c0_g1_i1|m.  NLVPRRPDKHRT                          
A5_TRINITY_DN102218_c5_g6_i2|m  ------------                          
A5_TRINITY_DN103684_c0_g2_i1|m  ------------                          
A5_TRINITY_DN103684_c0_g5_i1|m  ------------                          
A5_TRINITY_DN103684_c1_g3_i3|m  ------------                          
A5_TRINITY_DN34162_c0_g1_i1|m.  ------------                          
A5_TRINITY_DN81309_c2_g1_i1|m.  ------------                          
A5_TRINITY_DN81309_c2_g2_i2|m.  ------------                          
A5_TRINITY_DN88739_c0_g1_i1|m.  ------------                          
A5_TRINITY_DN89190_c1_g7_i1|m.  ------------                          
A5_TRINITY_DN93741_c1_g1_i1|m.  ------------                          
A5_TRINITY_DN97327_c1_g3_i2|m.  ------------                          
A8_TRINITY_DN28752_c0_g1_i1|m.  ------------                          
A8_TRINITY_DN33033_c0_g1_i1|m.  ------------                          
A8_TRINITY_DN40046_c1_g1_i1|m.  ------------                          
A8_TRINITY_DN42043_c0_g1_i1|m.  ------------                          
A8_TRINITY_DN49523_c2_g1_i1|m.  ------------                          
A8_TRINITY_DN52955_c1_g4_i1|m.  ------------                          
A8_TRINITY_DN52955_c1_g5_i1|m.  ------------                          
A8_TRINITY_DN52955_c1_g6_i1|m.  ------------                          
