# Supplementary material for: De novo transcriptome assembly analysis of weed Apera spica-venti from seven tissues and growth stages
Source: BMC Genomics. 2017 Feb 6;18:128. doi: 10.1186/s12864-017-3538-4 (PMC5294808; doi:10.1186/s12864-017-3538-4)
Supplement: Additional file 6: — Alignment of GSTF1 protein sequences from Apera and Alopecurus. (RTF 159 kb) [file 12864_2017_3538_MOESM6_ESM.rtf]

Additional file 6. Alignment of GSTF1 protein sequences from Apera and Alopecurus. 

                                         10        20        30        40        50        60        70        80        90       100                  
                                ....|....|....|....|....|....|....|....|....|....|....|....|....|....|....|....|....|....|....|....|
M7ZER1|M7ZER1_TRIUA             -------------------MGTEVKVKVFGPARSTCVARVLVCLEEVGAEYELVHVHLPAGDHKGPAHLARTPFGQVPAFQDGDLILFESRAISRYVLRK 
Q9ZS17|Q9ZS17_ALOMY Glutathion  ----------------------MAPVKVFGPAMSTNVARVTLCLEEVGAEYEVVNIDFNTMEHKSPEHLARNPFGQIPAFQDGDLLLWESRAISKYVLRK 
A2_GSTF1                        HLPPSFIPPPCSIQPAKGRGRVMSPMKVFGPARSTGVARVLICLEEVGAEYELVHVHIPAGEQKSPAHVARNPFGQVPAFQDGDLVLFESRAISKYILRK 
A3_TRINITY_DN89646_c0_g1_i2|m.  ---------------------------------------------EVGAEYELVHVHIPAGEQKSPAHVARNPFGQVPAFQDGDLVLFESRAISKYILRK 
A4_TRINITY_DN86114_c0_g1_i2|m.  ----------------------------------------------------------------------------------------ESRAISKYILRK 
A8_TRINITY_DN62165_c0_g1_i1|m.  -----------------------------------GVARVLICLEEVGAEYELVHVHIPAGEQKSPAHVARNPFGQVPAFQDGDLVLFESRAISKYILRK 
A7_TRINITY_DN61809_c0_g1_i1|m.  --------------------------------------------EEVGAEYELVHVHIPAGEQKSPAHVARNPFGQVPAFQDGDLVLFESRAISKYILRK 

                                        110       120       130       140       150       160       170       180       190       200         
                                ....|....|....|....|....|....|....|....|....|....|....|....|....|....|....|....|....|....|....|....|
M7ZER1|M7ZER1_TRIUA             GAS---DLLRENSLSKSTMVDAWLEAESHNFDRAMSAITFQCFVVPMFMGGTADERVVKENLEKLKVTLGVYEERLSRFKYLAGDFISLADLSHCPMAHY 
Q9ZS17|Q9ZS17_ALOMY Glutathion  YKTDEVDLLRESNLEEAAMVDVWTEVDAHTYNPALSPIVYQCLFNPMMRGLPTDEKVVAESLEKLKKVLEVYEARLSKHSYLAGDFVSFADLNHFPYTFY 
A2_GSTF1                        HAS---DLLKESSVSDSTMVDVWLEVESQKFDTIMCVITFQCFVVPIFMGGTTDDKIVNESLDKLGEVFKVYEARLSKSNYLAGEFISLADLSHAPMLHL 
A3_TRINITY_DN89646_c0_g1_i2|m.  HAS---DLLKESSVSDSTMVDVWLEVESQKFDTIMCVITFQCFVVPIFMGGTTDDKIVNESLDKLGEVFKVYEARLSKSNYLAGEFISLADLSHAPMLHL 
A4_TRINITY_DN86114_c0_g1_i2|m.  HAF---DLLKESSVSDSTMVDVWLEVESQKFDTIMCVITFQCFVVPIFMGGTTDDKIVNESLDKLGEVFKVYEARLSKSNYLAGEFISLADLSHAPMLHL 
A8_TRINITY_DN62165_c0_g1_i1|m.  HAS---DLLKESSVSDSTMVDVWLEVESQKFDTIMCVITFQCFVVPIF---------------------------------------------------- 
A7_TRINITY_DN61809_c0_g1_i1|m.  HAS---DLLKESSVSDSTMVDVWLEVESQKFDTIMCVITFQCFVVPIFMGGTTDDKIVNESLDKLGEVFKVYEARLSKSNYLAGEFISLADLSHAPMLHL 

                                        210       220       230       240   
                                ....|....|....|....|....|....|....|....|.
M7ZER1|M7ZER1_TRIUA             LLASPCSSVLDAYPRVKAWVDGMMDRPSVKKVMELMDAP-- 
Q9ZS17|Q9ZS17_ALOMY Glutathion  FMATPHAALFDSYPHVKAWWDRLMARPAVKKIAATMVPPKA 
A2_GSTF1                        LLVTPHAPMLNRYPHVKSWISGLMDRPSVKKVTELMDAP-- 
A3_TRINITY_DN89646_c0_g1_i2|m.  LLVTPHAPMLNRYPHVKSWISGLMDRPSVKKVTELMDAP-- 
A4_TRINITY_DN86114_c0_g1_i2|m.  LWVTPHAPMLNRYPHVKSWISGLMDRPSVKKVTELMDAP-- 
A8_TRINITY_DN62165_c0_g1_i1|m.  ----------------------------------------- 
A7_TRINITY_DN61809_c0_g1_i1|m.  LLVTPHAPMLNRYPHVKSWISGLMDRPSVKKVTELMDAP-- 
